# Disease-modifying effects of a glial-targeted inducible nitric oxide synthase inhibitor (1400W) in mixed-sex cohorts of a rat soman (GD) model of epilepsy

**DOI:** 10.1186/s12974-023-02847-1

**Published:** 2023-07-12

**Authors:** Suraj S. Vasanthi, Nikhil S. Rao, Manikandan Samidurai, Nyzil Massey, Christina Meyer, Meghan Gage, Mihir Kharate, Aida Almanza, Logan Wachter, Candide Mafuta, Lily Trevino, Adriana M. Carlo, Elijah Bryant, Brooke E. Corson, Morgan Wohlgemuth, Morgan Ostrander, Lucas Showman, Chong Wang, Thimmasettappa Thippeswamy

**Affiliations:** 1 Department of Biomedical Sciences, College of Veterinary Medicine, Iowa State University, Ames, 50011 USA; 2 W.M. Keck Metabolomics Research Laboratory, College of Veterinary Medicine, Iowa State University, Ames, 50011 USA; 3 Department of Veterinary Diagnostic and Production Animal Medicine and Statistics, College of Veterinary Medicine, Iowa State University, Ames, 50011 USA

**Keywords:** Nerve agents, Status epilepticus, Disease-modification, Telemetry, Behavioral dysfunction, Spontaneous seizures

## Abstract

**Background:**

Acute exposure to seizurogenic organophosphate (OP) nerve agents (OPNA) such as diisopropylfluorophosphate (DFP) or soman (GD), at high concentrations, induce immediate *status epilepticus* (SE), reactive gliosis, neurodegeneration, and epileptogenesis as a consequence. Medical countermeasures (MCMs—atropine, oximes, benzodiazepines), if administered in < 20 min of OPNA exposure, can control acute symptoms and mortality. However, MCMs alone are inadequate to prevent OPNA-induced brain injury and behavioral dysfunction in survivors. We have previously shown that OPNA exposure-induced SE increases the production of inducible nitric oxide synthase (iNOS) in glial cells in both short- and long- terms. Treating with a water soluble and highly selective iNOS inhibitor, 1400W, for 3 days significantly reduced OPNA-induced brain changes in those animals that had mild–moderate SE in the rat DFP model. However, such mitigating effects and the mechanisms of 1400W are unknown in a highly volatile nerve agent GD exposure.

**Methods:**

Mixed-sex cohort of adult Sprague Dawley rats were exposed to GD (132 μg/kg, s.c.) and immediately treated with atropine (2 mg/kg, i.m) and HI-6 (125 mg/kg, i.m.). Severity of seizures were quantified for an hour and treated with midazolam (3 mg/kg, i.m.). An hour post-midazolam, 1400W (20 mg/kg, i.m.) or vehicle was administered daily for 2 weeks. After behavioral testing and EEG acquisition, animals were euthanized at 3.5 months post-GD. Brains were processed for neuroinflammatory and neurodegeneration markers. Serum and CSF were used for nitrooxidative and proinflammatory cytokines assays.

**Results:**

We demonstrate a significant long-term (3.5 months post-soman) disease-modifying effect of 1400W in animals that had severe SE for > 20 min of continuous convulsive seizures. 1400W significantly reduced GD-induced motor and cognitive dysfunction; nitrooxidative stress (nitrite, ROS; increased GSH: GSSG); proinflammatory cytokines in the serum and some in the cerebrospinal fluid (CSF); epileptiform spikes and spontaneously recurring seizures (SRS) in males; reactive gliosis (GFAP + C3 and IBA1 + CD68-positive glia) as a measure of neuroinflammation, and neurodegeneration (especially parvalbumin-positive neurons) in some brain regions.

**Conclusion:**

These findings demonstrate the long-term disease-modifying effects of a glial-targeted iNOS inhibitor, 1400W, in a rat GD model by modulating reactive gliosis, neurodegeneration (parvalbumin-positive neurons), and neuronal hyperexcitability.

**Supplementary Information:**

The online version contains supplementary material available at 10.1186/s12974-023-02847-1.

## Introduction

Seizurogenic organophosphate (OP) nerve agents (OPNA) induce *status epilepticus* (SE) within 8 min of exposure [1, 2]. OPNA inhibits carboxyl ester hydrolases, particularly the acetylcholinesterase (AChE) to cause cholinergic crisis. Choline acts on the muscarinic and nicotinic receptors and induces classic parasympathetic (cholinergic) toxicity symptoms such as salivation, lacrimation, urination, and defecation (SLUD), tremors and muscle fasciculations [3, 4]. In the brain, AChE inhibition induces seizures. Medical countermeasures (MCMs), if administered immediately, control cholinergic symptoms and SE, to some extent, and prevent mortality. MCMs for OPNA toxicity include atropine sulfate, an oxime HI-6 dimethanesulfonate (DMS), and a benzodiazepine-derivative diazepam or midazolam. Atropine sulfate is a competitive, reversible antagonist of muscarinic receptors that prevents excessive glandular secretions, smooth muscle relaxation, and increases heart rate. HI-6 DMS is an asymmetric bis-pyridinium aldoxime and it is required for the reactivation of AChE before the enzyme ages. Therefore, both atropine and HI-6 should be administered immediately after an OPNA exposure. Experimental models suggest that diazepam or midazolam are effective in controlling behavioral SE and can reduce mortality if it is given in < 40 min of OPNA exposure [5–9]. Therefore, MCMs efficacy in OPNA toxicity is time sensitive.

Although MCMs control the acute symptoms of OPNA toxicity, they do not prevent SE-induced long-term brain injury [10–13]. Therefore, our hypothesis was that a combination of MCMs and a disease-modifying agent as an adjunct therapy that targets brain pathology will prevent SE-induced long-term brain injury in the soman model. Our previous study demonstrated a significant increase in the inducible nitric oxide synthase (iNOS) in the brains of OPNA exposed adult rats, which persisted for long-term in glial cells [14]. Treatment with a highly selective iNOS inhibitor, 1400W hydrochloride, suppressed serum nitrite production and also reduced iNOS levels in the brain [14, 15]. In addition, we found a significant increase in neuronal nitric oxide synthase (nNOS) post-SE, but unlike iNOS, it was only transiently upregulated [16, 17]. Excessive production of NO is toxic [18, 19]. Our previous studies in both rat kainate and DFP models of epilepsy demonstrated a significant disease-modifying effect when the animals were treated with 1400W for the first three days of post-SE [14, 20]. Although DFP is considered a surrogate for soman and structurally similar, the latter is a highly volatile agent. Like DFP, the onset of severe SE (convulsive seizures) in soman model is rapid and persistent [1, 2]. Our recent findings suggest that the initial SE severity determines the extent of brain injury in the OPNA model [21]. Therefore, rigorous SE quantification (behavioral and EEG based), grouping based on the SE severity, and randomization will minimize bias while testing the efficacy of disease-modifying drugs. We have thoroughly characterized and quantified SE severity in both DFP and soman models in adult male and female rats with or without telemetry devices [1, 2]. In this study, we demonstrate significant disease-modifying effect of 1400W in both males and females when mixed-sex cohorts were treated for 2 weeks post-MCM treatment following acute exposure to soman (GD). We demonstrate sex differences in EEG, behavioral outcomes, and histological changes (reactive gliosis and neurodegeneration) in the brain of animals exposed to soman and treated with 1400W or vehicle.

## Methods

### Animal source, care, and ethics statement

The adult male and female Sprague–Dawley rats (7–8 weeks old; 250–300 g; Charles River, USA) were used in the study. Males and females were housed in the same room but in individual cages with a 12-h light:dark cycle in an enriched environment. The experiments were conducted after 2–3 days of acclimatization as per the approved protocols by the Institutional Animal Care and Use Committees (IACUC protocol: 20-090) and complied with the NIH ARRIVE Guidelines for the Care and Use of Laboratory Animals. Soman exposure was done at MRI Global, Kansas City, MO, and all other experiments were conducted at the Iowa State University. All animals were euthanized at the end of the study with pentobarbital sodium and phenytoin sodium (100 mg/kg, i.p.) as per the American Veterinary Medical Associations Guidelines for euthanasia.

### Chemicals and reagents

The MRI Global, Kansas City, MO, United States acquired and administered the soman (> 95% pure by GC–MS) to the animals as per the approved IACUC protocols. Soman was diluted in cold 0.1 M PBS just before administration. HI-6 (99.9% pure- by LC/MS, Kalexsyn, Kalamazoo, MI, United States) and Atropine sulfate monohydrate (ATS, 99.9% pure by LC–MS, Tokyo Chemical Industry, USA) was diluted in saline at 250 mg/ml and 5 mg/ml, respectively. All key chemicals, except soman were validated by LC/GC–MS/MS, the chemicals purity and identity were authenticated before testing in animals. Midazolam (MDZ, prepared as 5 mg/ml stock solution) was supplied by the MRI Global. 1400W dihydrochloride (≥ 99% pure by HPLC, 98% by LC–MS, Tocris Bioscience, Bristol, UK) was diluted in distilled water at 20 mg/ml. Euthanasia solution (pentobarbital sodium and phenytoin sodium) was purchased from the Pharmacy at the ISU Lloyd Veterinary Medical Center. The antibodies used in this study are listed in Additional file 1: Table S1.

### Experimental design

**Fig. 1:**
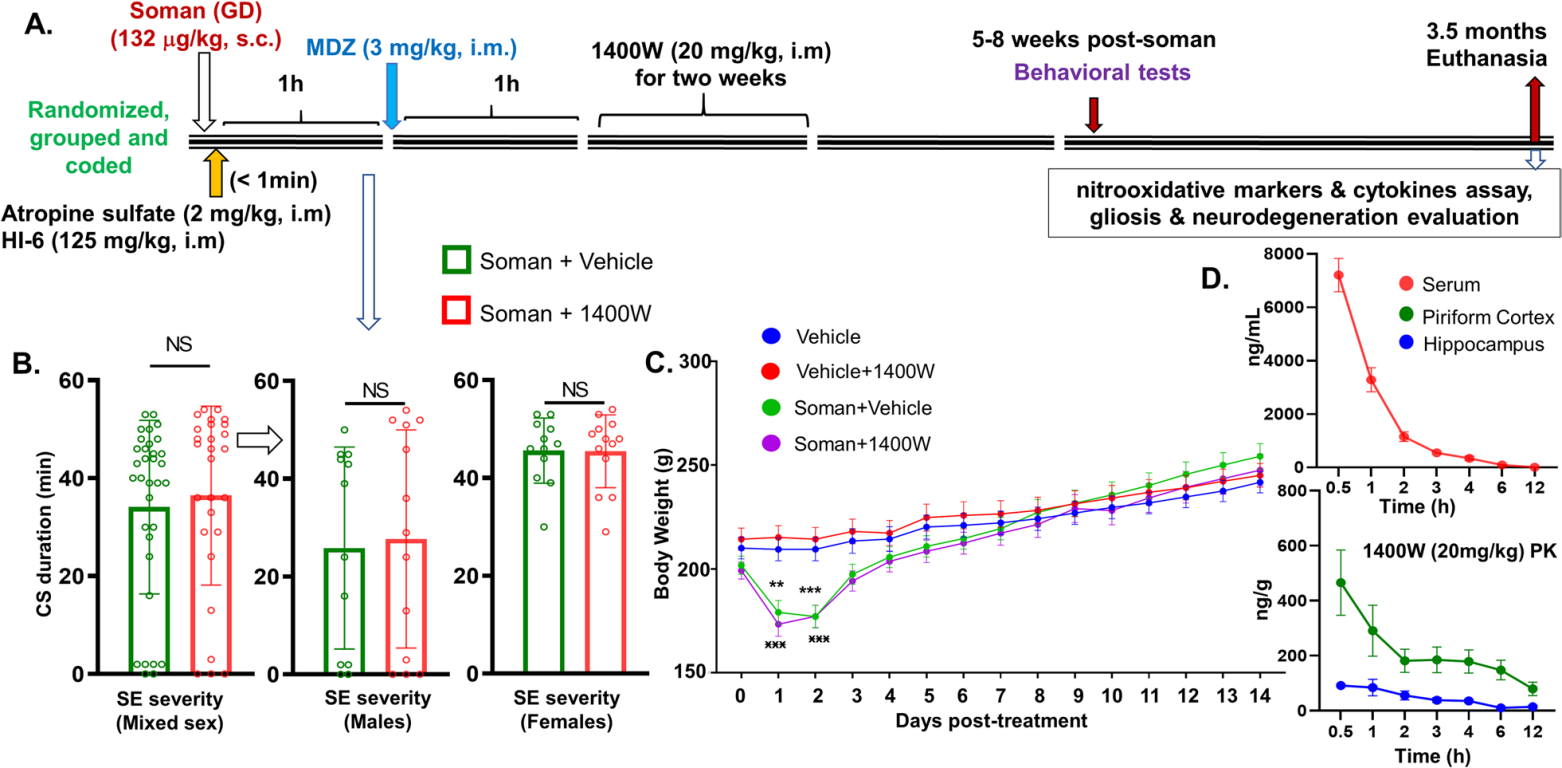
**A** Experimental design, animals grouping based on the initial SE severity (**B**), and bodyweight comparison post-exposure/treatment (**C**). There were no significant differences in SE severity between the vehicle and 1400W treated groups in either mixed-sex cohort or males or females (**B**). There was a significant weight loss in soman-exposed animals, irrespective of sex, during the first 3 days of post-soman exposure (**C**). 1400W concentrations in the serum, piriform cortex, and hippocampus of adult rats when the drug was administered as a single intramuscular injection at 20 mg/kg (**D**). (**B** Mixed-sex) Mann–Whitney test, *n* = 27–33; (**B** Males) Mann–Whitney test, *n* = 12–13; (**B** Females) Unpaired *t*-test, *n* = 12–13. **C** Repeated measures two-way ANOVA (Šídák’s multiple comparison), *n* = 24–26 for mixed-sex cohort (12–13/sex). **p < 0.01, ***^, **xxx**^*p* < 0.001 vs control. CS, convulsive seizures; NS, non-significant

The experimental designs are illustrated in Figs. 1A and 9A.

#### Experimental groups

We used 139 mixed-sex adult rats in this study. Animals were randomized, grouped, and coded/blinded. The non-telemetry experimental groups were: vehicle + vehicle (control, *n* = 24, 12/sex), vehicle + 1400W (*n* = 26, 13/sex), soman + vehicle (*n* = 27; 13 females, 14 males), and soman + 1400W (*n* = 30, 14 females, 16 males). These non-telemetry groups were used for behavioral testing. SE-severity matched mixed-sex cohorts (*n* = 6–10) from these groups were used for serum assays and brain immunohistochemistry. The tissues from the remaining animals were rapidly frozen in liquid nitrogen and archived for other mechanistic studies. We also used a subset of non-telemetry animals for brain structural and functional MRI acquisition after completing all behavioral testing, but the MRI data are not included in this communication. The telemetry experimental group were soman + vehicle (*n* = 16, 8/sex) and soman + 1400W (*n* = 16, 8/sex). These telemetered animals were used to acquire continuous video-EEG only in this study.

#### Telemetry device implantation prior to soman exposure

36 rats (18/sex) were implanted with CTA-F40 telemetry devices (Data Science International, MN, USA) for integrated video-EEG acquisition. The surgical procedure was carried out about 2 weeks before challenging with soman. Four animals were excluded from the study due to transmitter device-related issues during the 4 months study. The devices were implanted as described in our previous publications [2, 14, 21]. The animals were administered an analgesic buprenorphine (0.3 mg/kg, s.c.) before anesthetizing with 3.0% isoflurane (at 1L/min O_2_) and maintained at 1.0–1.5% during surgery. SomnoFlo small animal anesthetic machine by Kent Scientific (Torrington, CT, USA) was used for anesthesia. Artificial tears ointment was applied to the eyes to protect the cornea. A midline-incision was made on the skin of the head and the underlying tissue was resected to expose the parietal bone. Electrodes were implanted bilaterally on the dura mater of the cortical hemisphere by drilling holes into the skull. The telemetry device was implanted in a subcutaneous pouch in the flank region. The electrodes were secured to the skull with a dental cement (A-M Systems, Carlsberg, WA, USA) and the wound was closed with sterile clips. A triple antibiotic ointment (Vetropolycin) was applied to the wound, Baytril (enrofloxacin, 5 mg/kg, s.c., Bayer Pharmaceuticals, Pittsburgh, PA, USA) and 1 ml of 5% dextrose saline were administered subcutaneously. After full recovery, the animals were individually housed in a cage on PhysioTel receiver pads (RPC-1) which were connected to the Data Exchange Matrix 2.0 (DSI) for continuous video-EEG acquisition with Ponemah Software (DSI). Baseline video-EEG was recorded for 2 day–night cycles before exposure to soman.

#### Soman exposure and medical countermeasures

36 rats with telemetry devices and 57 rats without telemetry devices were exposed to soman as described in our recent publication [1]. Rats were treated with vehicle (phosphate buffered saline; PBS) or soman (GD, 132 µg/kg, s.c. 1.2 × LD_50_), followed by HI-6 (125 mg/kg, i.m.) and atropine sulfate (2 mg/kg, i.m.) within 1 min of soman exposure to control for peripheral cholinergic effects and mortality. The anticonvulsant midazolam (3 mg/kg, i.m.) was administered 1-h post-soman exposure to control behavioral SE.

#### Behavioral and EEG-based seizures quantification, SE severity-based grouping and coding, and 1400W or the vehicle treatment

Behavioral seizures were scored immediately after the animals were exposed to soman until the midazolam was administered, which is typically 60 min. Two experimenters (per animal) scored the seizures during SE directly. For telemetry animals, both behavioral and EEG-based seizure quantification were considered. Animals of both sexes were further grouped based on the similar SE severity and coded. The stages of seizures (stage 1 to stage 5) during SE in soman model, the stage-specific symptoms, and SE severity quantification are described in our recent publication [1]. Briefly, stage 1—excessive salivation, lacrimation, urination and defecation (SLUD), mastication, chewing; stage 2—the stage 1 symptoms progressed—tremors, wet-dog shakes, head nodding/neck jerks, kyphosis, and opisthotonos; stage 3—forelimb clonus, Straub tail, rearing and rigid extension of forelimbs; stage 4—repeated rearing, continuous forelimb clonus and loss of righting reflex; and stage 5—abducted limbs clonus and generalized seizures. One group received 1400W (20 mg/kg, i.m) and the other group received the vehicle (distilled water, i.m). 1400W or the vehicle was given twice daily for the first 3 days followed by a single injection per day for the next 11 days. The vehicle group received equal number and volumes of vehicle injections (i.m.). Animals that had < 20 min of convulsive seizures (stages 3–5) were considered under mild SE group.

#### Epileptiform spike rate and SRS quantification

Baseline EEG prior to soman exposure was used to normalize the post-soman EEG traces for accurate seizure and epileptiform spikes detection. Artifacts from electrical noise, grooming and exploratory behavior were identified and excluded from the epileptiform spike analysis as described in our publications [2, 20, 21]. We used NeuroScore version 3.4.0 software (DSI) for seizure detection and epileptiform spike quantification. However, all convulsive spontaneous recurrent seizures (SRS) detected by the NeuroScore were manually verified with the integrated video (behavioral seizure) and the power spectral changes. SRS on EEG was characterized by high-frequency and high-amplitude epileptiform spikes. Epileptiform spikes comprised interictal spikes with an amplitude greater than 340.41 ± 23.73 µV but less than 1100 µV. The epileptiform spike counts included the spikes within seizures that were automatically calculated by the NeuroScore. The epileptiform spikes were differentiated from artifact spikes based on amplitude, duration, interspike intervals, and spike rate as described previously [22]. The epileptiform spikes and seizure reports were generated for each animal for graphing and statistical analyses.

### Behavioral testing

Four groups from the non-telemetry cohort were used for all behavioral tests: two control groups (control and vehicle + 1400W) and two soman-exposed groups (soman + vehicle and soman + 1400W). All experiments were conducted by the same experimenters and started at the same time each day in a distraction-free environment. The room temperature and lighting were kept the same for all animals and for all experiments. All animals were handled gently to minimize stress. The animals from all four groups were randomized irrespective of sex and treatment. Equipment was thoroughly cleaned with 70% ethanol to remove urine or feces smell from the previous animal and dried before a new animal was introduced. All behavioral tests were conducted between 5 and 7 weeks post-soman exposure in the following order, as described below, starting with least aversive or less stressful to most aversive/stressful testing condition. Animals were given 2–3 days of rest between each new behavioral test.

#### Novel object recognition (NOR) and open field tests

The NOR test is a behavioral assay to measure learning and memory in rodents. The open field test, using the same apparatus, measures anxiety-like behavior. The test was conducted for 3 days: habituation day/open field test, testing day, and probing day. A low-wattage red light was used to illuminate the arena [23]. On the habituation day/open field test, animals were allowed to explore the arena (40 × 40 inches) for 10 min with no objects. The time spent at the center versus periphery of the arena was used to interpret the open field test data. On the day of testing, two similar objects (Stoelting Co., USA) were placed diagonally in the arena and the rat was released in the corner opposite to that of the objects and left to explore both objects for 5 min. Three hours or 24 h after testing (probing day), one of the two objects were replaced with a novel object and the rat was allowed to explore the objects for 5 min. The rats that explored less than 20 s were excluded from the analysis [24]. The movement of the rat was tracked by the camera operated by ANY-Maze software (Stoelting Co., USA). The time spent by the rat in the center versus periphery during the habituation phase and between the novel versus the familiar objects (discrimination index, DI = (t^novel^ − t^familiar^)/(t^novel^ + t^familiar^)) were analyzed and compared between the groups and sexes.

#### Elevated zero/circular maze test

The elevated zero/circular maze test assesses anxiety-related behavior. In contrast to the open field, the elevated circular platform (47-in. diameter) was set at a height of 20 inches off the ground. The circular platform (4-in. wide) was divided into two enclosed arenas, with 12-in.-high walls, opposite to each other and two open arenas between the enclosed arenas. The rats were placed in at the junction of open and closed arenas and allowed to explore the maze for 5 min. Movement of the rat was monitored remotely via an overhead camera operated by ANY-Maze software (Stoelting Co., USA) connected to a computer in a separate room. The percentage time spent by the rat in open versus closed quadrants between each group and sex were quantified and analyzed.

#### Accelerated rotarod test

Animals were tested for motor learning and motor coordination using an accelerating rotarod apparatus (AccuRotor 4-channel, Omnitech Electronics Inc. and Start Fusion v6.2 AccuRotor Edition Software). We used a modified protocol from our previous publications [14, 21]. The acceleration speed was set from 5 to 60 rpm with a duration of 180 s. The test was conducted for 3 days with 2 days of training and a day of testing with a 24-h rest between training and testing. Evaluation of motor coordination and learning was done in three trials on both training and testing days, and the mean time spent by the rats on the rotating rod (latency to fall) was measured and compared between the groups and sex. The rats that jumped off the apparatus repeatedly were excluded from the analysis.

#### Contextual and cued fear conditioning test

The rats were tested for contextual and cued fear conditioning to assess their ability to recall an association between a conditioned stimulus (a tone) and an aversive unconditioned stimulus (an electric foot shock). The test consisted of conditioning (day 1) and probing (day 2). On the day of conditioning, the rats were allowed to explore the apparatus for 2 min, followed by an auditory tone (70–80 dB) for 20 s. At the last 2 s of the auditory tone, a 0.7-mA foot shock was delivered continuously. The conditioning paradigm was repeated four times per session with an inter-trial interval of 80 s, followed by a rest for 80 s. The rats were probed 24 h after the conditioning session. The probing protocol was the same as conditioning test but without a foot shock. The movement of the rat was tracked by the camera operated by ANY-maze software (Stoelting Co., USA). The freezing time during conditioning and probing were recorded and analyzed and compared between the groups and sexes. The freezing time (%) increase was calculated by averaging the freezing times (%) from all four inter-trial intervals (ITIs) and subtracting these from the freezing time (%) during habituation phase of the probe test.

### Euthanasia and tissue collection

All animals were euthanized with a lethal dose of pentobarbital sodium and phenytoin sodium (100 mg/kg, i.p.) at the end of the study. Some animals were perfuse-fixed with 4% paraformaldehyde (PFA) and the brains were isolated and processed for immunohistochemistry (IHC) from non-telemetered animals. The blood and CSF were collected for nitrooxidative markers and cytokine assays from non-telemetered animals. The blood was centrifuged at 1000–1500 × g for 10 min to separate serum. Both serum and CSF were aliquoted and stored at – 80 °C until further analysis. The tissue samples collected from telemetry groups were archived for future studies.

### Tissue processing, immunohistochemistry, imaging, and cell quantification

After perfusion, the brains were isolated and stored in 4% PFA for 24 h and transferred to 25% sucrose in PBS for at least 3 days at 4 °C. The brains were then embedded in gelatin (15% Porcine gelatin Type A, 7.5% sucrose, and 0.1% sodium azide in PBS, Sigma, MO, USA) at 37 °C for 3 h and stored overnight at 4 °C. The gelatin blocks were then rapidly frozen in liquid nitrogen-cooled 2-methyl butane. The brains were cut on a cryostat at 16 μm thickness from rostral to caudal using a cryostat (ThermoFisher) as described in our previous publication [20]. Five sections per slide were collected on chrome alum gelatin (Pfalz and Bauer, CT, USA) coated slides and stored at − 20 °C until they were processed for IHC. Each slide contained representative sections from rostral to the caudal end of the brain.

The brain sections were subjected to antigen retrieval by treating with citrate buffer (10 mM citric acid and 0.05% Tween-20, pH 6.0) at 95 °C for 20 min. After cooling, the slides were covered with cover plates and transferred to Shandon racks for IHC. The sections were washed with PBS 3 times and incubated in blocking buffer for an hour (10% donkey serum, 0.05% TritonX-100 in PBS) at room temperature. The sections were incubated with primary antibodies (GFAP-mouse/IBA1-Goat/NeuN-rabbit/C3-rat/CD68-rabbit/PVB-rabbit) overnight at 4 °C. The next day, slides were washed 3 times in PBS and incubated with appropriate secondary antibodies (AlexaFluor 488 anti-mouse/ AlexaFluor 488 anti-rabbit/ Rhodamine Red™-X anti-goat/ Rhodamine Red™-X anti-rabbit/ Biotinylated anti-rat) for an hour at room temperature. The dilutions for antibodies, the source, and the species in which the antibodies were raised are included in Additional file 1: Table S1. Slides were again washed 3 times in PBS, rinsed in water to remove salts, and mounted with VectaShield^®^ mounting medium containing DAPI nuclear stain (Vector Laboratories Inc., CA, USA). The slides were stored at -4 °C until imaging.

To investigate the extent of neurodegeneration, the brain sections were processed for NeuN-Fluro Jade B (FJB) dual staining. The sections were first stained for NeuN using the procedure described above. The sections were then washed with PBS three times and distilled water before immersing in freshly prepared 0.006% potassium permanganate for 5 min. The slides were washed in distilled water two times for 2 min to remove potassium permanganate residues. The slides were incubated in freshly prepared 0.0003% FJB solution in 0.1% acetic acid for 10 min in the dark, followed by three washes in distilled water for 1 min. The slides were air dried overnight in the dark and mounted with Surgipath acrytol (Leica Biosystems, IL, USA).

The sections were imaged using the Leica DMi8 inverted fluorescence microscope (Wetzlar, Germany) fitted with Leica K5 passive cooled sCMOS camera system. Images from 3 coronal sections per brain from each group, representing the same regions of the brain from rostral to caudal were used for cell quantification. IBA1, GFAP, FJB-positive cells and C3 + GFAP colocalized cells were quantified manually using the multipoint tool in ImageJ software as described in our previous publication [14, 16]. Reactive astrocytes and microglia were also quantified manually. The morphology-based parameters were large cell bodies, short and thick processes in reactive phenotypes in contrast to a small cell body, thin and long processes in homeostatic-state controls [25–28]. All quantifications were blinded till the analyses were completed. In addition, we first did morphology-based GFAP + ve cell quantification only, followed by GFAP + C3 quantification which was also blinded. Likewise, for reactive microglia. CD68 + IBA1 colocalized foci were quantified using the analyze particles function in ImageJ.

### Diaminobenzidine (DAB)-NeuN staining and stereology

The DAB-NeuN immunostaining was performed to identify the neurons in the hilus of the DG, CA1, and AMY + PC regions for stereological counting. After processing the brain sections for NeuN primary and secondary biotinylated antibodies, the slides were washed with PBS (3×). The horse radish peroxidase-avidin solution (HRP, Vectastain) was prepared fresh and the sections were incubated at RT for an hour. After PBS washing (3×), the slides were placed in a DAB solution (25 mg DAB, 50 ml PBS, and 250 µl hydrogen peroxide) until desired stain intensity was observed. The slides were washed in tap water for 10 min, air-dried, and mounted with acrytol. A Zeiss Axio Imager 2 light microscope was used to image the sections and neuronal counts were performed using the Micro Bright Field Bioscience Stereo Investigator v9 software (Williston, VT, USA). The neurons were quantified at a 40 × objective with the following parameters—counting frame 70 × 70, SRS grid 150 × 150, and defined detector 0-7-0.

### Cytokines and chemokine assays

We used RayBiotech^®^ ELISA kits (Norcross, GA, USA) for key proinflammatory cytokine and chemokine assays (IL1-β, IL-6, MCP-1, TNFα). Pilot testing of previously confirmed positive samples reassured the quality of the kits. The serum was used without dilution, but the CSF was used at 1:2 dilution. The assay was conducted according to the manufacturer’s protocol in duplicates along with the positive and negative controls. Using the standard reagents, the standard curve for each cytokine was obtained independently which was used to determine the concentrations of cytokines/chemokine in the test samples with the absorbance read at 450 nm and expressed as pg/mL.

### Glutathione assay

The GSH and GSSG (oxidized GSH) were determined in the serum using a ThermoScientific^®^ Glutathione Colorimetric Detection Kit (Carlsbad, CA, USA). The assay was performed according to the manufacturer’s protocol. GSH and GSSG assays were done in duplicates and previously known positive and negative controls were used simultaneously alongside the test samples. To measure oxidized glutathione (GSSG), 2-vinylpyridine (2VP) treated standards and samples were used. The concentration of GSSG was half of the GSH concentration read from the curve (i.e., 1 GSSG = 2 GSH). Free/reduced glutathione (GSH) concentrations were determined by subtracting the GSSG concentrations from the values obtained from the samples and standards that were treated with 2VP. Absorbance was read at 405 nm. Concentrations were expressed as μM.

### Nitrite assay

The serum nitrite assay was determined using a Sigma-Aldrich^®^ Nitrite Assay or Griess Reagent Kit (St. Louis, MO, USA) according to the manufacturer’s protocol. The assay was done in duplicates and previously known positive and negative controls were included simultaneously. A standard curve was prepared using the standard reagents. The standard curve was used to derive the concentrations of nitrite in the test samples with the absorbance read at 540 nm and the nitrite concentrations were expressed as μM.

### ROS assay

We used OxiSelect™ Intracellular ROS Assay Kit (Green Fluorescence) to determine ROS levels in the serum. The assay was performed as per the manufacturer’s protocol. The ROS content in unknown samples was analyzed by comparing with the predetermined DCF standard curve with the absorbance read at 480 nm excitation/530 nm emission. The ROS concentration was expressed in relative fluorescence units (RFUs).

### Liquid chromatography–tandem mass spectrometry (LC–MS/MS) quantification of 1400W in the serum and brain tissue

#### Extraction of 1400W from serum and brain tissue and LC–MS/MS sample preparation

Serum (25–75 µL) and brain tissue (15–60 mg) samples were used for the analysis. Prior to sample extraction, internal standard was added to each still frozen sample, 50 µL of 0.2 mM homophenylalanine (AL0-7500, Phenomenex, Torrance, CA). Extractions were begun with the addition of 0.2 mL of methanol to all serum and tissues samples. The tissue samples were homogenized using a Bead Mill 24 Homogenizer (Thermo Fisher Scientific, Inc., Waltham, MA). 0.8 mL of water was added to all samples before sonication for 5 min via a sonicating water bath. Samples were vortexed for 5 min prior to centrifugation at 16,300 g for 10 min. The extract supernatants were combined for each sample in 2-mL glass autosampler vials and dried in a speed-vac concentrator for 10 h. The dried extracts were resuspended in 600 µL of 1:1:2 methanol:water:acetonitrile before being filtered with 0.2-micron centrifugal filters (Cat. No. UFC30LG25, Millipore Sigma, Burlington, MA) before being subjected to LC–MS/MS analysis.

A 1400W standard curve with a range of 0.25–2000 ng per sample was prepared as 1:1 serial dilution in 20% methanol before being extracted and subjected to LC–MS/MS analysis in the same manner as the biological samples. Pharmacokinetic analysis was determined using PK solver^®^ software [29].

#### 1400W LC–MS/MS quantification

For LC–MS/MS 1400W quantification, the liquid chromatography separations were performed with an Agilent Technologies 1290 Infinity II UHPLC instrument equipped with an Agilent ZORBAX RRHD HILIC Plus analytical column (2.1 mm × 100 mm, 1.8 µm) that was coupled to a 6470 triple quadrupole mass spectrometer with an atmospheric-pressure chemical ionization (APCI) source (Agilent Technologies, Santa Clara, CA). The chromatography was carried out at 40 °C with a flow rate of 0.400 mL/min. Data evaluation and peak quantitation were performed using Agilent MassHunter Qualitative Analysis (version 10.0) and Agilent MassHunter Quantitative Analysis (version 10.0) software (Agilent Technologies, Santa Clara, CA). Target peaks were found at 3.5 min retention time for 1400W and 3.25 min for the internal standard. 1400W quantification was finally determined by relative abundance to the internal standard and 1400W standard curve, before being made relative to the measured sample mass and volumes.

### Statistical analysis

We used GraphPad Prism 9.0 for statistical analysis. Normality tests were performed using the Shapiro–Wilk test, where applicable. Based on the normality of the data, *t*-test or Mann–Whitney test was applied for comparing data between two groups. One-way ANOVA with appropriate post hoc test was used to compare multiple groups with one factor, while sex differences were tested using the two-way ANOVA to detect significant interactions between sex and treatment effects [30] with appropriate post hoc test for multiple comparisons across groups. If the interaction effects were found to be significant, then the data were presented as separate sex cohorts with relevant post hoc test for multiple comparisons. A mixed-effects model or repeated measures two-way ANOVA was performed to determine overall main treatment effects when comparing across different brain regions in immunohistochemistry panels. Further statistical details are included in the figure legends of the corresponding figures. The *p* values of the region-wise two-way ANOVA interaction tests for sex differences are listed in Additional file 1: Table S2.

### Rigor, sample size, and inclusion or exclusion criteria

All animals used in this study were purchased from the same source (Charles River, USA). Animals were first randomized ignoring sex, stage of the estrous cycle, and bodyweight. Age and sex-matched controls and the soman-exposed groups treated with vehicle or 1400W were blinded. The sample size and power were determined based on our previous OPNA studies outcome from gliosis and neurodegeneration parameters [21] at 95% confidence interval. SE severity matched males and females were used for IHC and biochemical assays. The exclusion criteria for each behavioral test analysis are included in respective Method section.

## Results

The detailed statistical tests for each result, sample size per group for each experiment, *p* value for sex interaction, and the type of statistical test applied for each data set are listed in Additional file 1: Table S2.

### Comparison of the initial SE severity in soman-exposed animals treated with vehicle or 1400W, the change in bodyweights during the treatment period, and pharmacokinetics of 1400W

Experimental design is illustrated in Fig. 1A. The soman-exposed animals were divided into two groups and the initial SE severity was compared before assigning the animals for vehicle or 1400W treatment. There were no significant differences in the SE severity between the vehicle and 1400W groups when they were analyzed as mixed-sex cohorts or as male or female groups independently (Fig. 1B). SE severity is the duration of convulsive seizures during the 60 min between soman exposure and midazolam injection. All soman-exposed animals, irrespective of sex or treated with vehicle or 1400W, significantly lost weight in the first 3–4 days of post-exposure, while there was no weight loss in both control groups (control; Vehicle + 1400W) (Fig. 1C). However, soman-exposed animals irrespective of sex, recovered their bodyweight and showed a similar trajectory as their respective control animals (Fig. 1C).

1400W was detected in the serum and brain tissue of adult rats (without soman exposure) for up to 12 h when the drug was administered as a single intramuscular injection at 20 mg/kg. In the serum, the peak concentration of 7.21 ± 0.62 µg/mL was observed at 30 min post-injection. The concentrations of 1400W were higher in the piriform cortex than the hippocampus (Fig. 1D).

### 1400W reduced the soman-induced motor, cognitive, and anxiety-like behavioral dysfunction

All behavioral tests were conducted between 5 and 8 weeks post-soman (Fig. 1A).

#### 1400W significantly reduced the soman-induced motor deficits

**Fig. 2:**
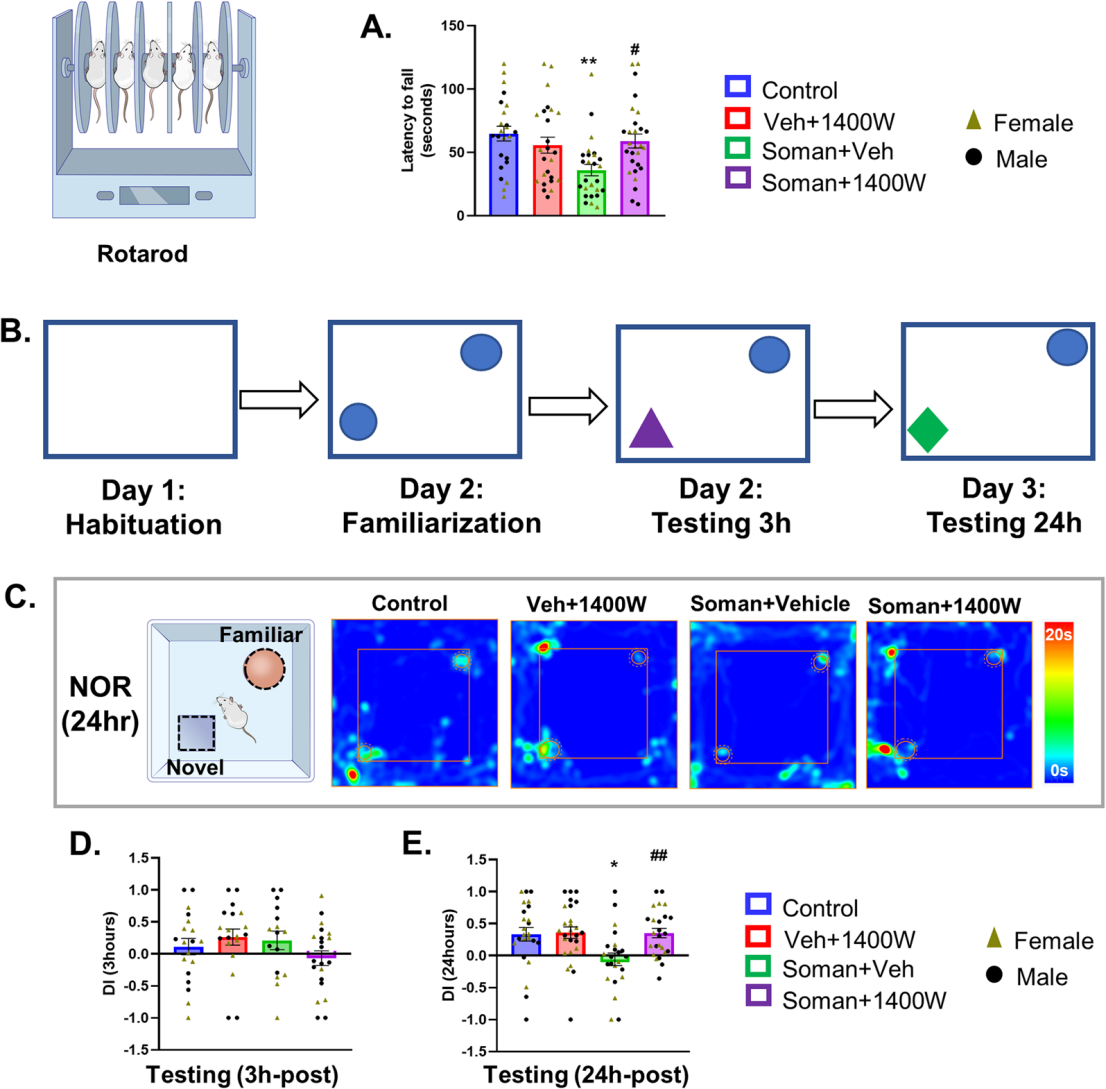
1400W significantly reduced soman-induced motor deficit, measured by rotarod, in mixed-sex cohort (**A**). Experimental design for the Novel Object Recognition (NOR) test (**B**) and representative heatmap from each group during 24 h probe testing (**C**). 1400W significantly rescued soman-induced long-term memory deficits at 24 h post-familiarization (**E**) but not at 3 h (**D**). No sex interaction was detected in either rotarod or NOR test either at 3 h or 24 h. **A, D, E** Two-way ANOVA (Tukey’s multiple comparisons test), *n* = 16–26 for mixed-sex cohort (8–13/sex). *p < 0.05, **p < 0.01 vs control; ^#^*p* < 0.05, ^##^*p* < 0.01 vs Soman + vehicle

Acute exposure to soman in the long-term caused a significant reduction in accelerated motor coordination in the vehicle-treated group, compared to controls, and 1400W mitigated the effect (Fig. 2A). No sex interaction was detected.

#### 1400W mitigated the soman-induced long-term memory deficit

A schematic diagram illustrating the experimental design for the Novel Object Recognition test (Fig. 2B) and representative heatmaps are shown in Fig. 2C. There were no significant differences in memory retention in any of the four groups when the animals were tested 3 h post-familiarization of the objects (Fig. 2D). However, when tested 24 h post-familiarization, the soman vehicle group showed a significant reduction in the discrimination index which was mitigated by 1400W (Fig. 2E). No sex differences were observed.

#### 1400W mitigated the soman-induced fear extinction-like behavior

**Fig. 3:**
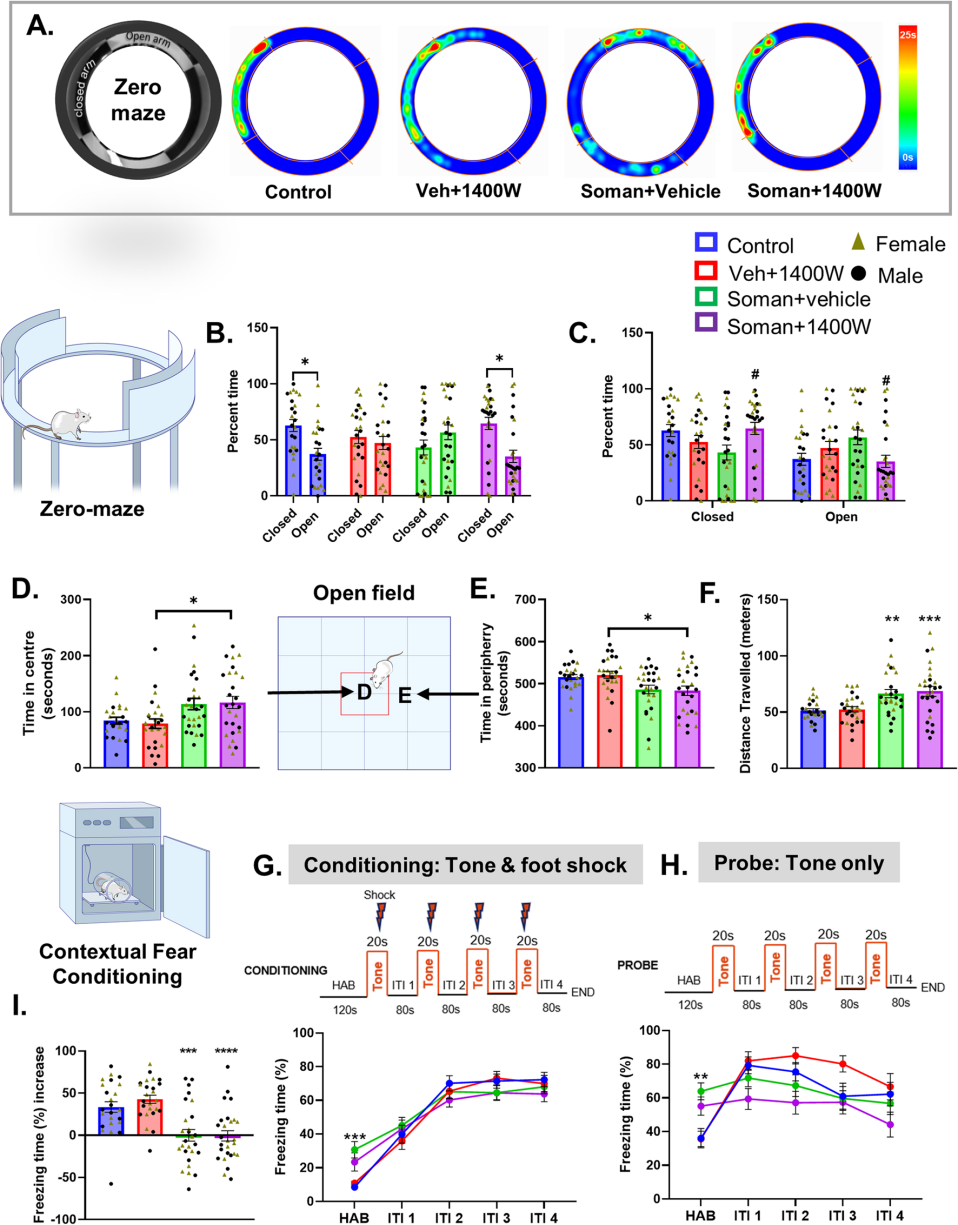
The elevated zero/circular maze (**A–C**) and open field tests (**D–F**). Representative heatmaps from each group is shown (**A**). Control and soman + 1400W groups significantly spent more time in closed quadrants (**B, C**). Soman exposed 1400W treated group spent significantly more time at the center (**D**) or less time in the periphery (**E**) in an open field (illustrated in the schematic diagram) than the vehicle 1400W group. Soman animals irrespective of treatment travelled more distances in the open field versus the controls (**F**). Contextual fear conditioning test (**G–I**). During habituation before conditioning (**G**) and probing (**H**), soman-exposed groups froze longer than the controls. There were no significant differences in freezing time during the inter-trial intervals (ITI) between any groups during the conditioning phase (**G**). Differences in freezing time emerged during the ITI during probing (**H**). Significant differences in percent freezing time increase were observed in soman-exposed animals (**I**). The percent freezing time of all inter-tone intervals, except habituation, was compared between groups in **I**. **B** Paired *t*-test (Control, VEH 1400W) and Wilcoxon matched-pairs signed rank test (Soman VEH, Soman 1400W). **C–F, I** Two-way ANOVA (Tukey’s multiple comparisons test), *n* = 11–15/sex; **G, H** two-way repeated measures ANOVA (Tukey’s multiple comparisons test), *n* = 24–27 for mixed-sex cohort, *n* = 11–15/sex. *p < 0.05, **p < 0.01, ***p < 0.001 vs respective control; ^#^*p* < 0.05 vs soman + vehicle

We have previously shown that acute exposure to DFP, an analog of soman, causes injury to the amygdala [21], the part of the brain involved in regulating fear/anxiety-like behavior. In the elevated zero maze test (Fig. 3A), the control and soman-exposed 1400W treated groups spent significantly less time in open quadrants compared to closed quadrants, while the soman-exposed vehicle-treated group spent relatively more time in open quadrants (Fig. 3B, C). However, in the open field test (Fig. 3D), soman-exposed groups, irrespective of vehicle or 1400W, in contrast to their respective controls, spent more time at the center of the open field chamber or less time in the periphery (Fig. 3D, E). The total distance traveled in the open field testing was significantly higher in soman-exposed animals irrespective of vehicle or 1400W treatment (Fig. 3F). No sex interaction was observed in either elevated zero maze or open field test.

#### Impact of soman and 1400W on contextual fear conditioning

When the animals were placed in the fear conditioning chamber (habituation), soman-exposed animals irrespective of vehicle or 1400W treatment froze significantly longer than their respective control groups (Fig. 3G, H). Interestingly, after habituation, all four groups responded similarly to the conditioning test that involved a tone for 20 s followed by foot shock for 2 s at the end of the tone (Fig. 3G). During the probe test habituation, 24 h after conditioning, soman-exposed animals also froze significantly longer than the controls (Fig. 3H). However, during the first inter-trial interval (i.e., between the introduction of the first tone without foot shock and the second tone), control animals froze for longer time than the soman-exposed groups (Fig. 3H). The freezing time decreased in subsequent inter-trial intervals in both control groups, more so in the control than the vehicle + 1400W group. Among the soman-exposed groups, the freezing time decreased after the last trial (Fig. 3H). Overall, soman-exposed animals showed a significant reduction in freezing time increase than their respective controls suggesting the compromised fear pathway (Fig. 3I). No sex interaction was observed.

### 1400W significantly reduced the soman-induced oxidative and nitrooxidative stress

**Fig. 4:**
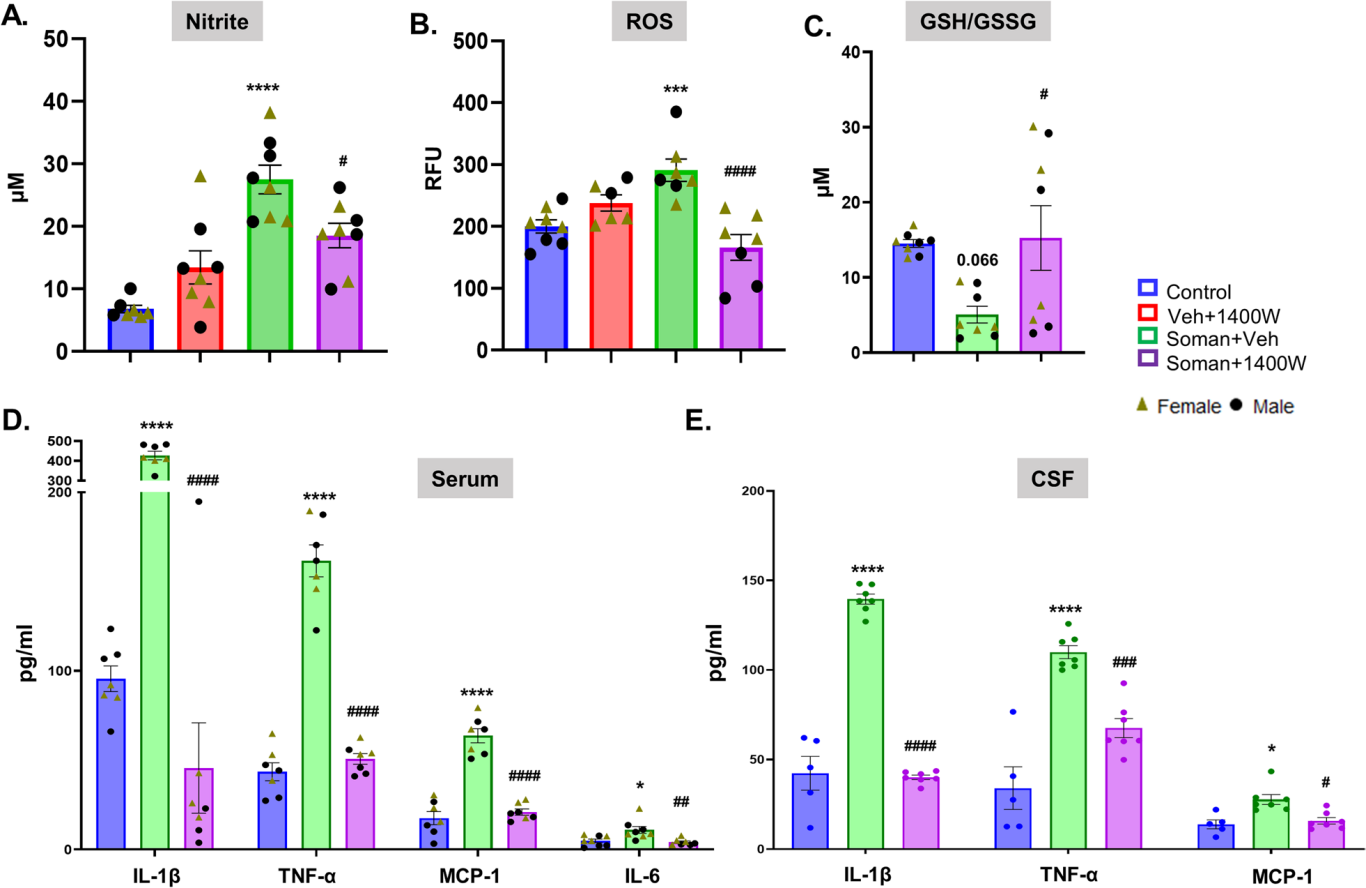
1400W significantly reduced the soman-induced nitrite (**A**) and ROS (**B**) production in the serum, and mitigated soman-induced glutathione oxidation (**C**). 1400W significantly reduced soman-induced proinflammatory cytokines (IL1β, TNFα) and a chemokine, MCP1 in both serum and CSF (**D, E**). Serum IL-6 was also affected by the treatment (**D**). Assays were performed at 3.5 months post-treatment. **A–D** Two-way ANOVA (Tukey’s multiple comparisons test); **E** one-way ANOVA (Tukey’s multiple comparison test for TNF-α and IL1-β) and one-way ANOVA (Dunn’s multiple comparison test for MCP-1); for all, *n* = 6–8 for serum; 5–7 for CSF; *p < 0.05, ***p < 0.001, ****p < 0.0001 vs control; ^#^*p* < 0.05, ^##^*p* < 0.01, ^###^*p* < 0.001, ^####^*p* < 0.0001, vs Soman + vehicle

Serum samples from 3.5 months post-soman animals, treated with or without 1400W, and age-matched vehicle control animals were used for nitrite, ROS, and glutathione assays. Soman exposure caused a significant increase in serum nitrite and ROS concentrations and 1400W significantly reduced the soman-induced effects (Fig. 4A, B). The ratio of reduced and oxidized glutathione (GSH: GSSG) was significantly reduced in the soman-exposed vehicle-treated group and 1400W mitigated the soman-induced effects (Fig. 4C).

### 1400W significantly reduced soman-induced proinflammatory cytokines in the serum and CSF

The serum and CSF samples were collected under terminal anesthesia, before the animals were perfused for brain histology, and used for the assay. Soman exposure significantly increased IL-1β, TNFα, and MCP1 in both serum and CSF, and 1400W significantly reduced soman-induced effects (Fig. 4D, E). Similar effects were observed in IL-6 levels in the serum (Fig. 4D).

### 1400W reduced the soman-induced reactive gliosis in several brain regions

**Fig. 5:**
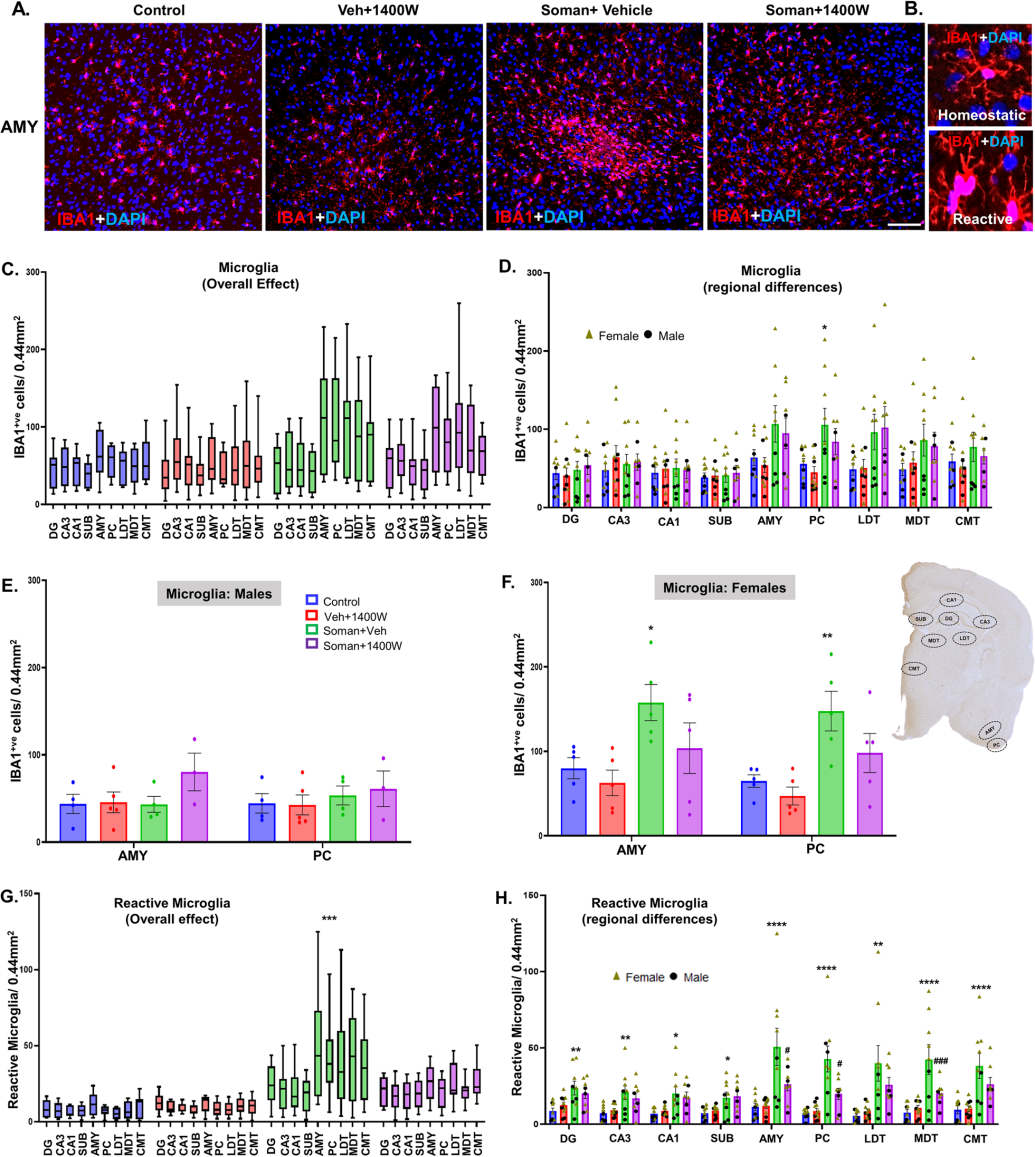

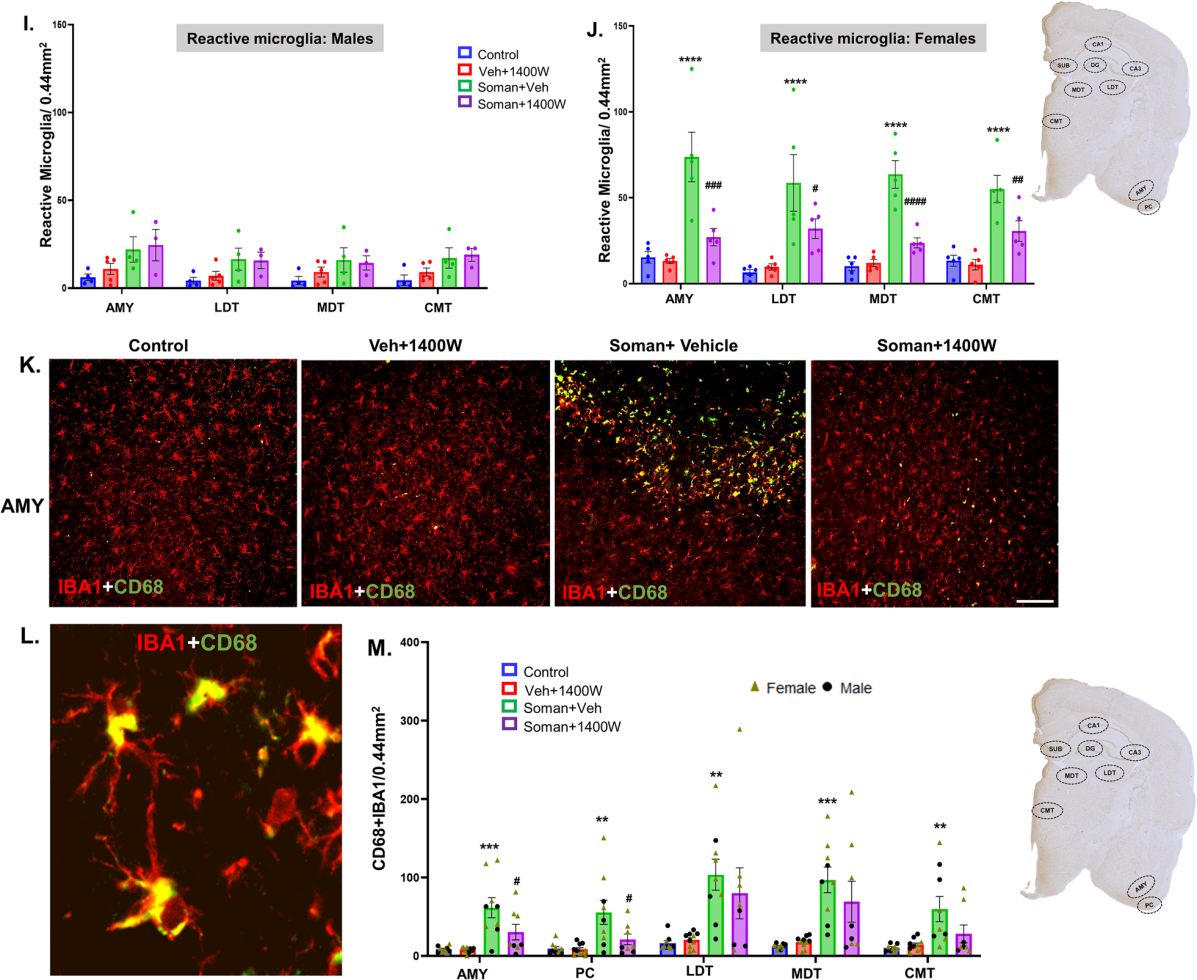
**A** Representative photomicrographs of the brain sections from female rats stained for IBA1 (microglia), and DAPI (nuclear stain); scale bar 100 µm. **B** Representative higher magnification images of reactive/homeostatic microglia. Microgliosis (**C–F**) and reactive microgliosis (**G–J**) quantification. Overall effect (**C, G**) and regional differences (**D, H**) in mixed sex, and differences in males (**E, I**) and females (**F, J**) are shown for microgliosis and reactive microgliosis. Differences in CD68 + IBA1 colocalization in AMY from different groups are shown (**K, L**). The brain regions where significant increase in CD68 + IBA1 colocalization were observed in soman-exposed animals were quantified and compared with other groups (**M**). **C, G** Two-way ANOVA (Tukey’s multiple comparisons test); **D–F, H–J, M** region-wise two-way ANOVA (Tukey’s multiple comparisons test). For all, *n* = 8–10 for mixed-sex cohort or 3–5/sex. *p < 0.05, **p < 0.01, ***p < 0.001, ****p < 0.0001 vs control; ^#^*p* < 0.05, ^##^*p* < 0.01, ^###^*p* < 0.001, ^####^*p* < 0.0001 vs soman + vehicle

We focused on cell counts in the hippocampal (DG, CA1, CA3, and subiculum) extrahippocampal piriform cortex (PC), amygdala (AMY) and the thalamic regions laterodorsal thalamic nucleus (LDT), mediodorsal thalamic nuclei (MDT), and centromedian thalamic nuclei (CMT). We counted IBA1-positive microglia (Fig. 5C–F), reactive microglia (morphology based; Fig. 5G–J), and CD68 + IBA1-positive colocalized foci (Fig. 5M). Representative photomicrographs of the AMY immunostained for IBA1 and CD68 from all four groups are shown in Fig. 5A, B and K, L.

There was an overall marginal increase in microgliosis in the extrahippocampal regions in soman-exposed animals (Fig. 5A, C, D). Interestingly, when region-wise comparison was made for each sex, the increase in the IBA1-positive cells were observed in both AMY and PC in females, but not in males (Fig. 5E, F). When the reactive microglia with large cell bodies and thick cytoplasmic processes (Fig. 5B) were counted, there was an overall effect, i.e., significant increase in reactive microglia in soman-exposed vehicle group (Fig. 5G). When regional differences were analyzed, significant differences were observed in all regions (Fig. 5H). In females, the significant differences between soman-exposed vehicle and 1400W groups was observed in the amygdala and all three thalamic regions (LDT, MDT, and CMT) (Fig. 5J), but not in males (Fig. 5I). Reactive microglia more frequently contain CD68 [31, 32]. Therefore, we further analyzed CD68 colocalized IBA1 cells (Fig. 5L). 1400W significantly reduced soman-induced effects in AMY and PC, while it was not significantly reduced in the thalamic nuclei (Fig. 5M). Interestingly, no sex difference was observed in CD68 + IBA1 cells in these regions.

**Fig. 6:**
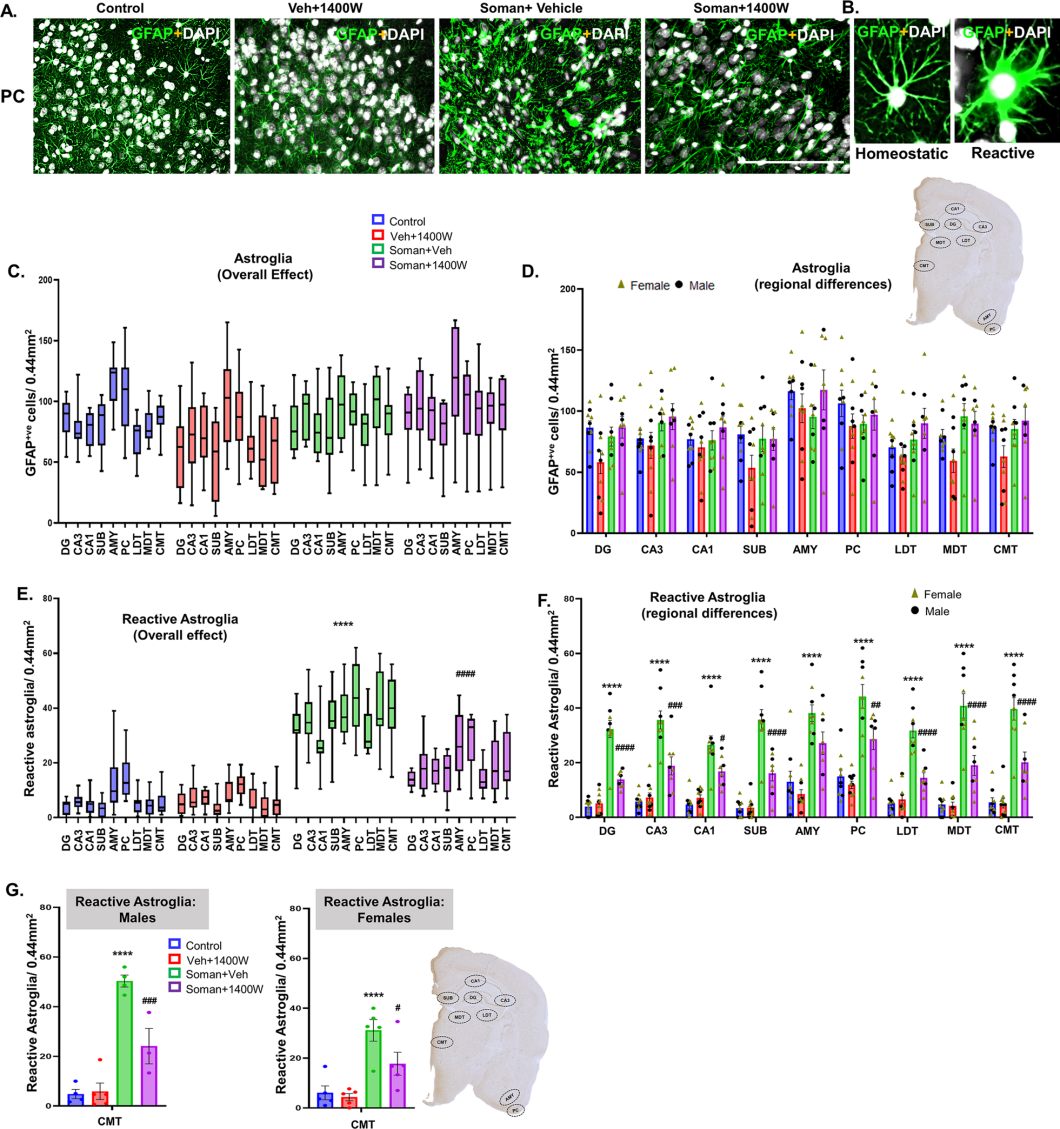

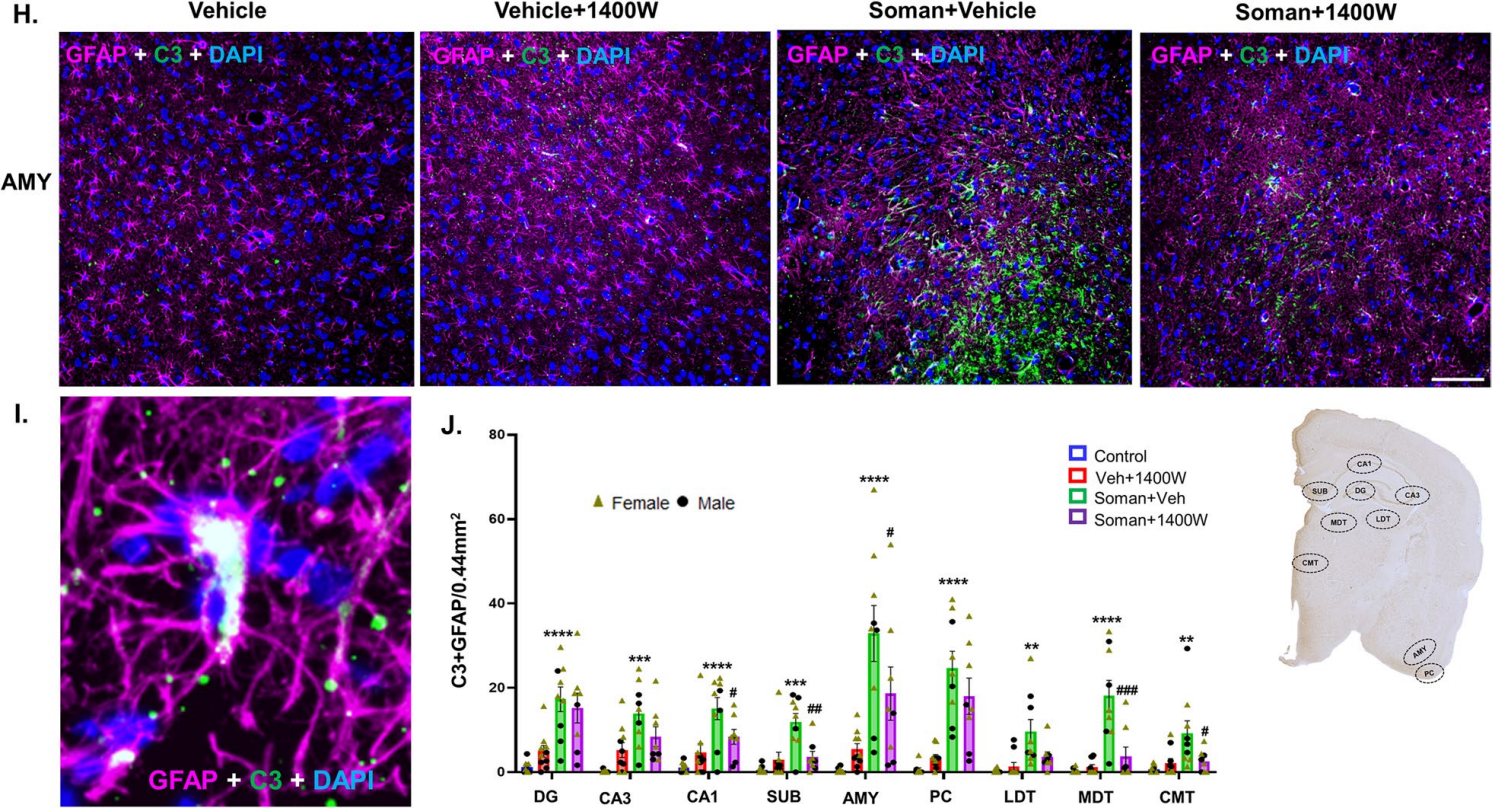
**A** Representative photomicrographs of the brain sections from female rats stained for GFAP (astrocytes), and DAPI (nuclear stain); scale bar 100 µm. **B** Representative higher magnification images of reactive/homeostatic astrocytes (green labeled cells). Astrogliosis (**C, D**) and reactive astrogliosis (**E, F**) quantification. Overall effect (**C, E**) and regional differences (**D, F**) in mixed sex are presented. Sex differences were observed only in the centromedial thalamic nuclei (CMT) (**G**) for reactive astrogliosis. Representative images of GFAP and C3-positive cells in AMY from each group is shown (**H, I**); scalebar 100 µm. Quantification of C3 containing GFAP-positive cells in other brain regions (**J**). **C, E** Two-way ANOVA (Tukey’s multiple comparisons test; **D, F, G, J** region-wise two-way ANOVA (Tukey’s multiple comparisons test). For all, *n* = 8–10 for mixed-sex cohort or 3–5/sex. **p < 0.01, ***p < 0.001 ****p < 0.0001 vs control; ^#^*p* < 0.05, ^##^*p* < 0.01, ^###^*p* < 0.001, ^####^*p* < 0.0001 vs soman + vehicle

Representative GFAP-positive astrocytes in piriform cortex from each treatment group are presented in Fig. 6A. There were no significant differences in astrogliosis per se in soman-exposed vehicle or 1400W treated groups in mixed-sex cohorts when analyzed using the repeated measures two-way ANOVA (overall treatment effect) or in the region-wise two-way ANOVA (Fig. 6C, D). There were no significant sex interactions in any region. However, when the reactive astrocytes with thick cytoplasmic process and large cell bodies with intense GFAP stained cells (Fig. 6B) were compared, there was a significant increase in the soman + vehicle group across all brain regions and 1400W significantly reduced the soman-induced effects (Fig. 6E, F). Sex interaction was observed only in CMT region (Fig. 6G). Since reactive astrocytes upregulate complement 3 (C3) [33, 34], we investigated C3-positive GFAP cells (Fig. 6H, I). There was a significant increase in such cells in soman + vehicle group across all regions, and 1400W significantly reduced C3 colocalized astrocytes in CA1, subiculum, AMY, MDT and CMT regions (Fig. 6J). There was no sex interaction. In this study, we adopted the two-step validation approach for reactive astrogliosis quantification; blinded morphology-based quantification and blinded GFAP + C3 colocalized astrocytes quantification. The results from both methods were largely complementary.

### Soman exposure caused significant neurodegeneration and 1400W mitigated the effect in some brain regions

**Fig. 7:**
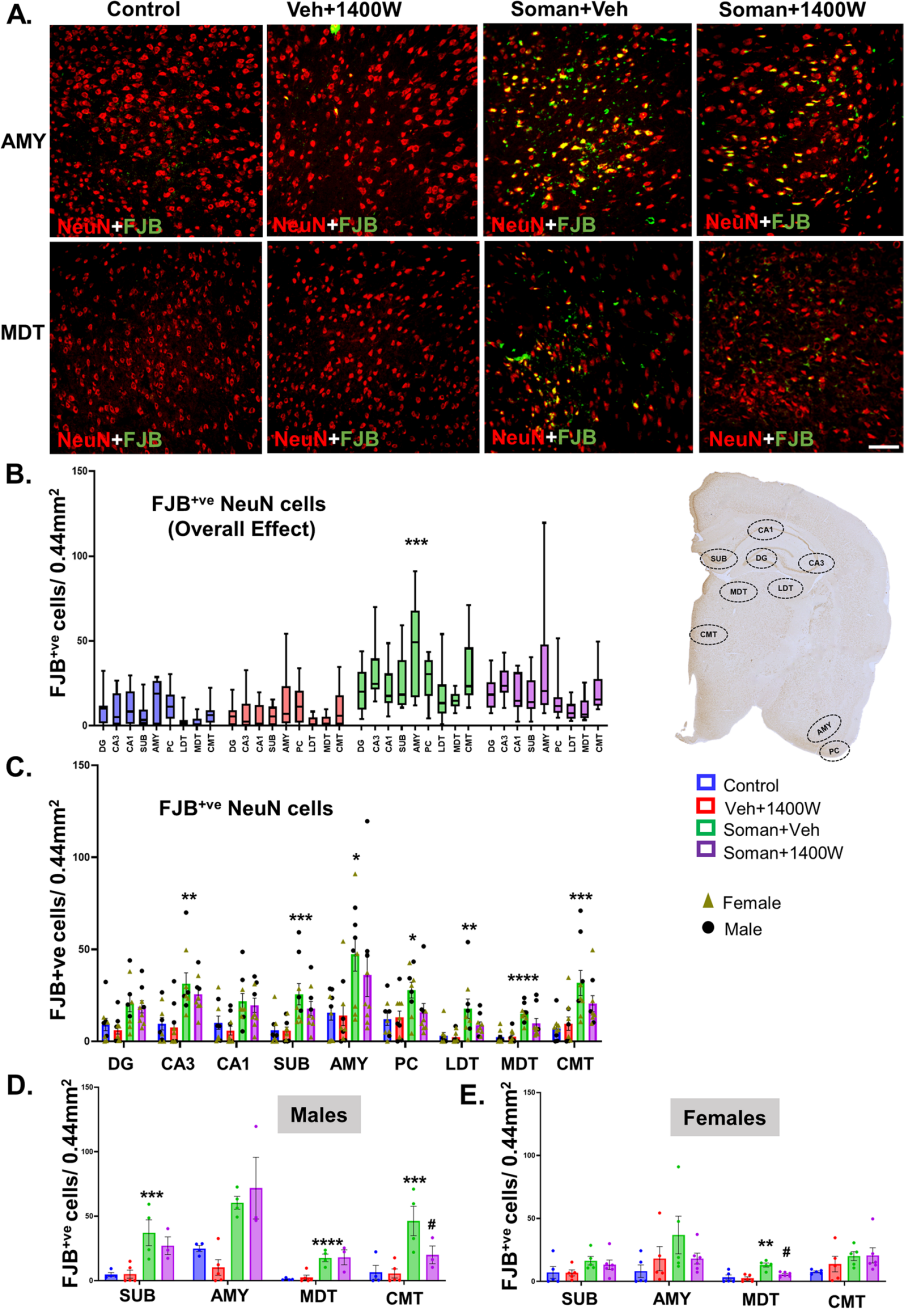
**A** Representative photomicrographs of the brain sections from female rats stained for NeuN (red) and FJB (green, co-label appears yellow). Amygdala (AMY) and mediodorsal thalamus (MDT); scalebar 100 µm. **B–E** cell quantification. Overall effect (**B**) and regional differences (**C**) in mixed sex are presented. Sex differences were observed in subiculum (SUB), amygdala (AMY), mediodorsal and centromedial thalamic nuclei (MDT, CMT). **B** Two-way ANOVA (Tukey’s multiple comparisons test); **C–E** region-wise two-way ANOVA (Tukey’s multiple comparisons test). For all, *n* = 9–10 for mixed-sex cohort or 3–6/sex. *p < 0.05, **p < 0.01, ***p < 0.001, ****p < 0.0001 vs control; ^#^*p* < 0.05 vs soman + vehicle

**Fig. 8:**
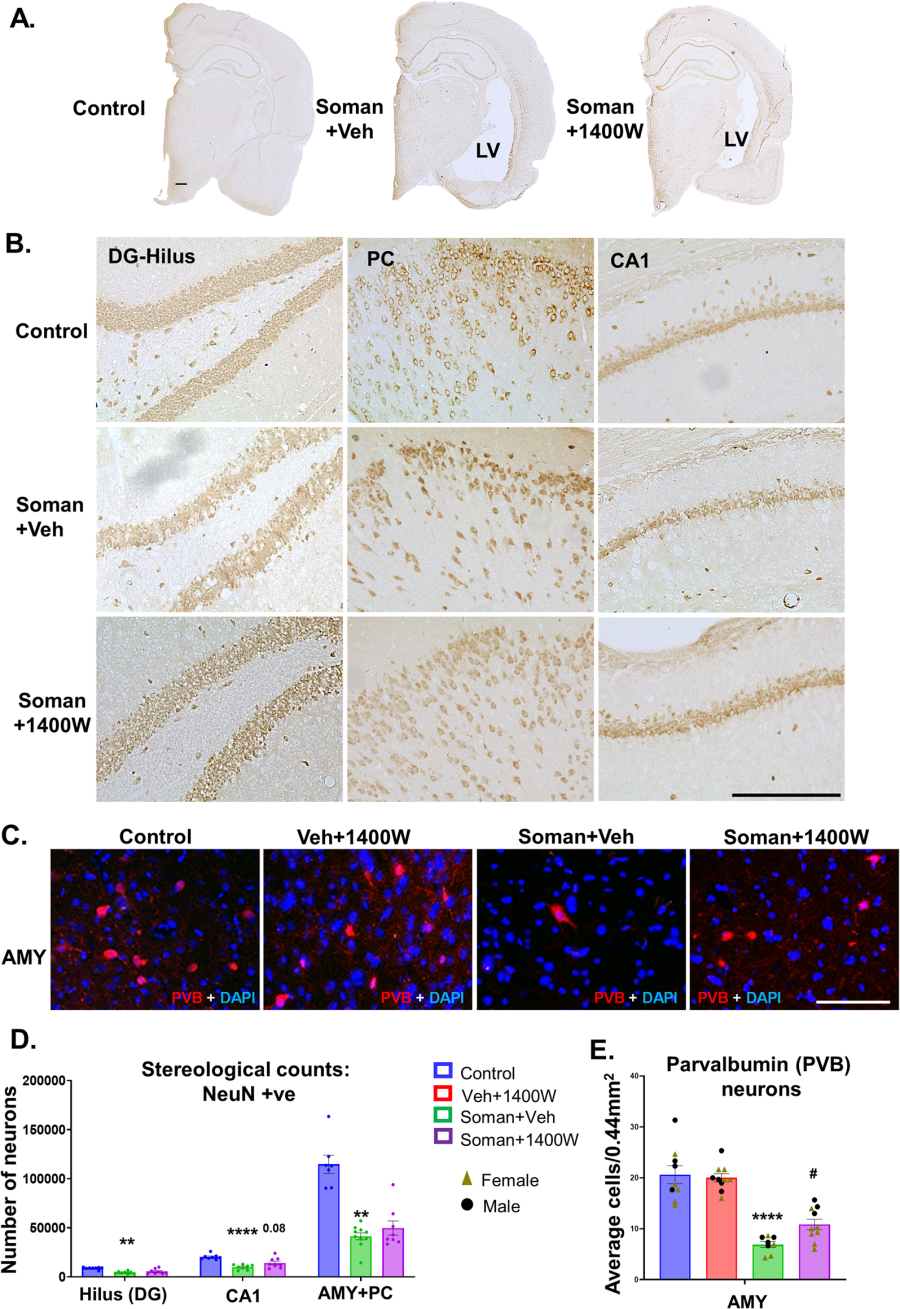
Representative brain sections used for stereological NeuN (DAB stained; **A, B**) and parvalbumin (PVB) immunostained images (**C**) are shown. Neuronal cell quantification, by stereology, from the hilus of the dentate gyrus, CA1 region of the hippocampus, amygdala and piriform cortex from female rats (**D**). PVB-positive neurons from AMY (**E**). **D** One-way ANOVA with multiple comparisons test, *n* = 7–10; **E** two-way ANOVA (Tukey’s multiple comparisons test), *n* = 8–10 (4–7 per sex). **p < 0.01, ****p < 0.0001 vs control; ^#^*p* < 0.05 vs soman + vehicle. Scalebar 500 μm (**A**), 50 µm (**B**), 100 µm (**C**)

Soman exposure has been known to induce long-lasting neurodegeneration in various parts of the brain [1, 35, 36]. Representative images of AMY and MDT are shown in Fig. 7A. FJB-positive NeuN cells were significantly increased in the soman-exposed vehicle-treated group (Fig. 7B). Regional analysis indicated a significant increase in all regions except dentate gyrus and CA1 (Fig. 7C). Sex interaction was observed in subiculum, AMY, MDT and CMT regions (Fig. 7D, E). 1400W significantly reduced the soman-induced neurodegeneration in MDT in females and CMT in males (Fig. 7D, E). We further quantified the absolute number of neurons in the hilus of the dentate gyrus, CA1, AMY and PC. Low magnification (4x) of whole half brain images revealed enlarged lateral ventricle in vehicle-treated soman-exposed animal compared to 1400W treated and control groups (Fig. 8A). Representative histological images are shown in Fig. 8A and B. We observed a significant reduction in the number of neurons in the hilus of the dentate gyrus, CA1 hippocampus, piriform–amygdala region in the soman-exposed animals (Fig. 8D). In the hilus, there was ~ 55% reduction in neurons in soman vehicle group while in the 1400W group there was ~ 35% loss compared to the control (Fig. 8D). In CA1, there was ~ 50% reduction in the number of NeuN positive neurons while in the 1400W treated group, there was ~ 25% reduction compared to control (Fig. 8D). In the amygdala and piriform cortex, there was > 50% neuronal loss in soman-exposed animals and the 1400W had no effect compared to control (Fig. 8D). We further analyzed a subset of inhibitory neurons, the parvalbumin (PVB) positive neurons in the most vulnerable brain region, the amygdala (Fig. 8C). 1400W significantly reduced soman-induced reduction of PVB neurons (Fig. 8E). No sex interaction was observed in stereological counts or in PVB neurons.

### 1400W significantly reduced soman-induced epileptiform spiking and SRS in males

**Fig. 9:**
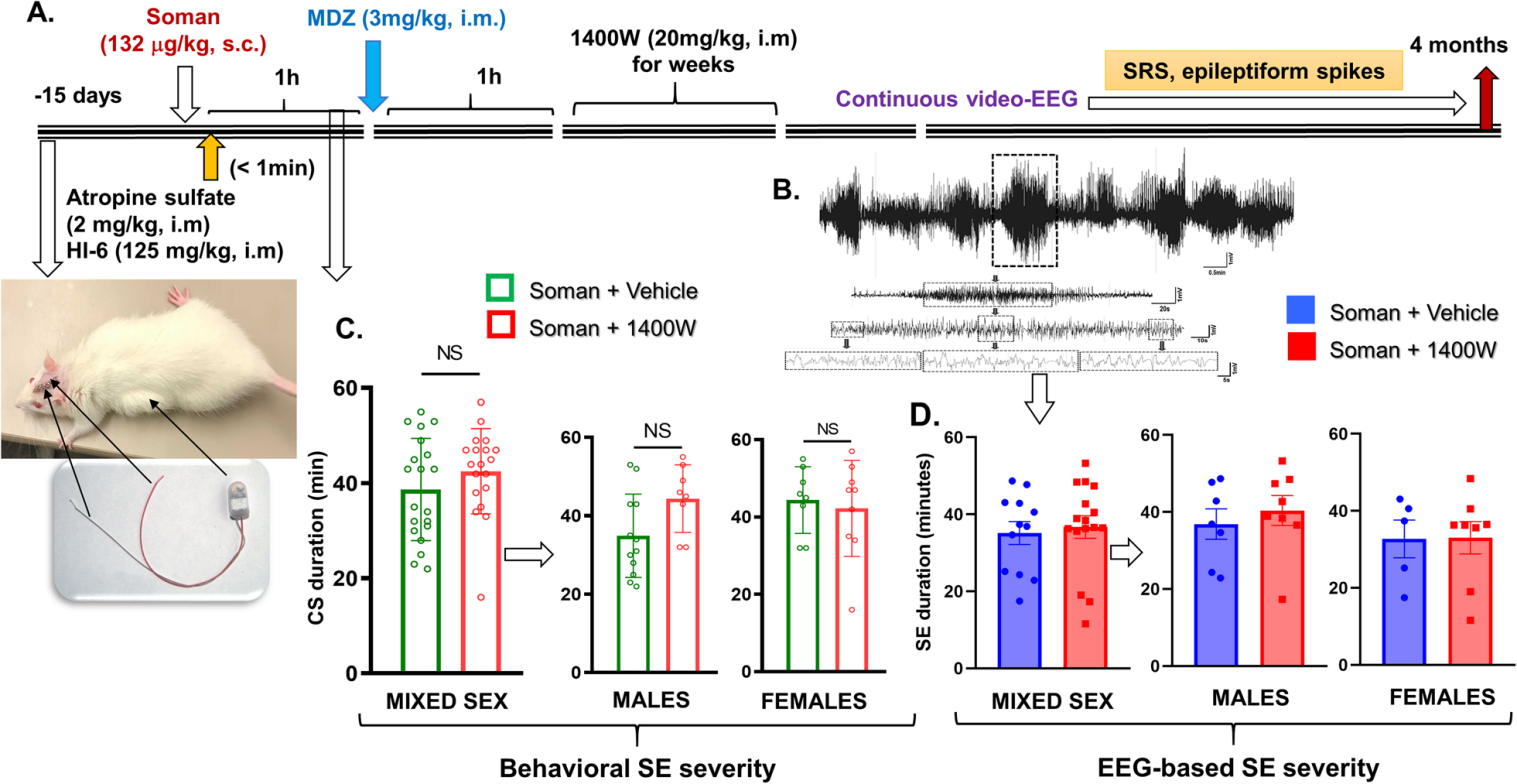
**A** Telemetry experimental design, initial SE severity quantification and grouping (**C, D**). A telemetry device was implanted 2 weeks before exposure to soman. **B** A representative EEG trace during SE is shown. **C** Animals were grouped based on the initial behavioral SE severity. There were no significant differences in SE severity between the vehicle and 1400W treated groups in either mixed-sex cohort or males or females (**C, D**). **C** Unpaired *t*-test, *n* = 19–20 for mixed-sex cohort (*n* = 8–12/sex); (**D** mixed-sex) Mann–Whitney test, *n* = 12–16; (**D** males–females) unpaired *t*-test, *n* = 5–8. NS, non-significant

**Fig. 10:**
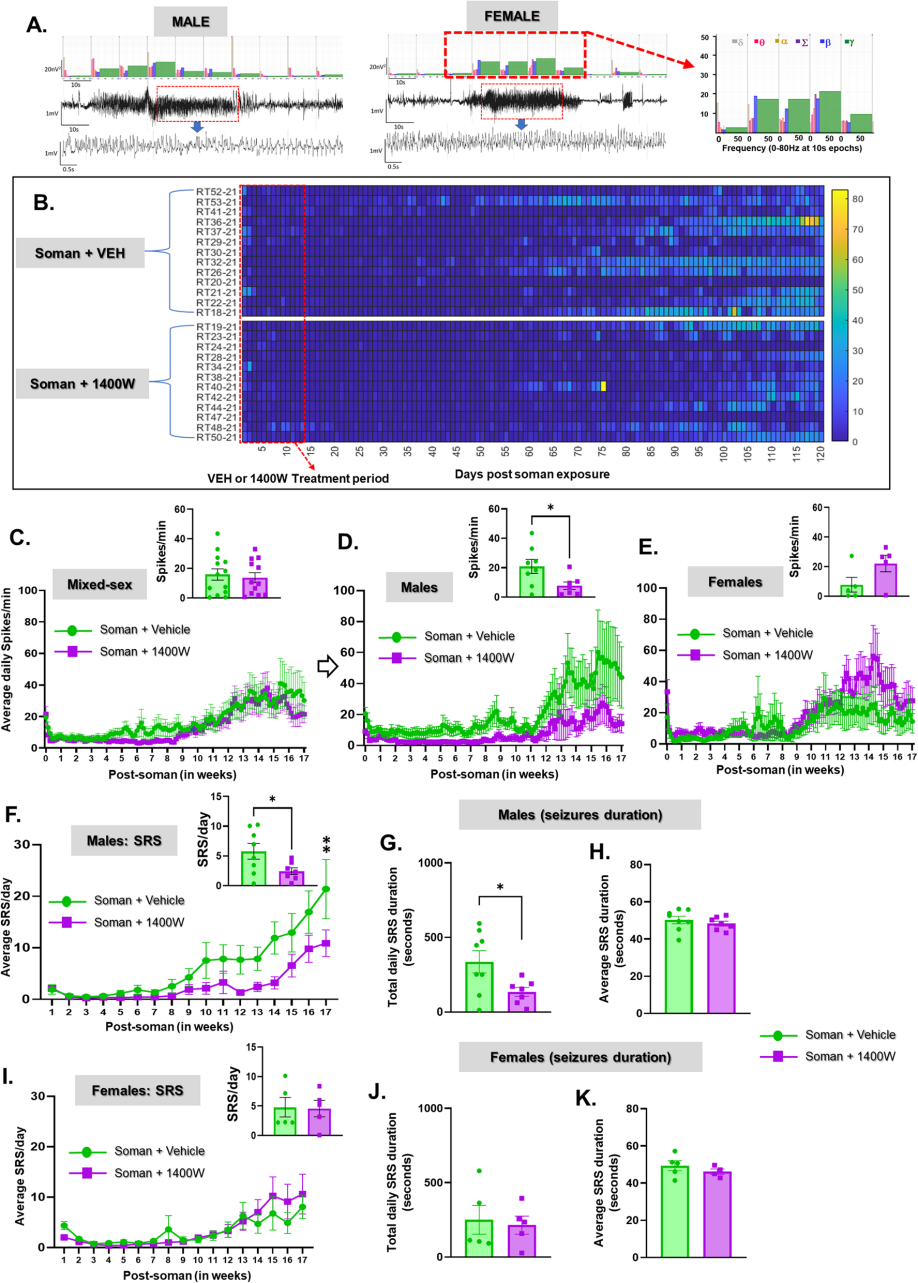
1400W treatment significantly reduced soman-induced epileptiform spikes and spontaneously recurring seizures (SRS) in males but not in females. **A** Representative EEG traces showing spontaneous recurrent seizures (SRS) in a male and a female rat and the corresponding gamma powerband elevation. **B** Heatmap of SRS episodes during the entire 4-month study period. **C**–**E** Epileptiform spike rate comparison between soman + vehicle and soman + 1400W (mixed-sex cohort—**C**; males—**D**, females—**E**). **F**–**K** SRS frequency, total seizure duration and average seizure duration in males and females compared between vehicle and 1400W treated soman-exposed animals. **C, D, F–K** Unpaired *t*-test; **E** Mann–Whitney test; **F, I** mixed-effects analysis (Šídák’s multiple comparisons test); for all, *n* = 12–13 for mixed-sex cohort or 5–8/sex; *p < 0.05, **p < 0.01

The experimental design for telemetry device implanted animals is illustrated in Fig. 9A. A representative EEG trace of a convulsive seizure (stage 5) during SE is shown in Fig. 9B. In telemetry-implanted cohorts, the differences between male and female behavioral SE for the same dose of soman as for non-telemetry cohort (Fig. 9C) were minimal but EEG-based SE severity was lower than behavioral SE severity (Fig. 9C, D). Irrespective of sex or stage of estrous cycle, there was no significant difference in the initial SE severity, neither in behavioral or EEG-based SE, between the vehicle and 1400W treated animals (Fig. 9C, D). Representative EEG traces from a male and a female rat showing a typical SRS episode during the long-term continuous video-EEG acquisition for 4 months are shown in Fig. 10A. A heatmap showing the frequency of SRS in the soman-exposed animals, treated with vehicle or 1400W, during the 4 months is illustrated in Fig. 10B. The EEG analysis revealed a significant and progressive increase in epileptiform spikes and SRS in soman-exposed vehicle-treated males, and 1400W significantly reduced the epileptiform spikes and SRS in males but not in females (Fig. 10C–F, I). Interestingly, in females, 1400W increased the epileptiform spikes during the last 4 weeks of the acquisition period, however, the differences were not statistically significant (Fig. 10E). The epileptiform spikes comprised interictal spikes and the spikes within seizures. 1400W also significantly reduced the total duration of time an animal spent seizing each day in the male rats but not in females (Fig. 10G, J). The average duration of individual seizure episodes was ~ 50 s, with no effect of 1400W treatment on the average duration of individual seizure episodes in either sex (Fig. 10H, K).

## Discussion

Chemoconvulsant-induced SE increases the production of reactive oxygen and nitrogen species in the brain [37–39]. The role of nitrooxidative stress in SE-induced epilepsy is emerging as a potential target for disease modification [14, 20, 40, 41]. Our previous short- and long-term studies in the rat DFP model revealed a significant upregulation of iNOS in the hippocampus and piriform cortex at 48 h and 7 days post-SE [14]. Immunohistochemistry of brain sections at 7d and 12 weeks post-SE confirmed the increased iNOS expression, predominantly in microglia, while nitrosylated proteins (3-NT) were observed in neurons in both the hippocampus and piriform cortex [14]. Treating with a highly selective iNOS inhibitor, 1400W, twice a day for the first three days of DFP exposure significantly reduced DFP-induced changes in the brain including the spontaneously recurring seizures in the 3-month study [14]. However, in this study, animals with severe SE were less responsive to the 1400W treatment in the DFP-induced behavioral dysfunction implying the dosing regimen used in the previous study was inadequate to mitigate behavioral dysfunction across all severity groups. Furthermore, the previous DFP study did not address sex differences. In the current soman study, we demonstrate the differential effects of soman and 1400W in the brains of both sexes and its impact on behavioral, EEG, and brain histological outcomes. We also demonstrate the long-term changes in the peripheral biomarkers of nitrooxidative stress and proinflammatory cytokines in the serum and CSF in response to acute exposure to soman and the effects of two weeks of 1400W treatment after soman exposure.

As per the NIH-NINDS guidelines for rigor [42], we designed and conducted experiments in age-matched mixed-sex cohorts. In SE-induced epilepsy studies, the initial seizure severity is a critical variable [1, 2, 21, 43]. In both DFP and soman models, we found that females had lower electrographic seizures (duration) than the observed behavioral seizures [1, 2]. Therefore, it is likely that in non-telemetered females, there is an exaggerated peripheral response to soman. We grouped the animals based on behavioral SE scores to balance between vehicle or 1400W treatment to detect the real effects of the interventional drug. Despite females having severe SE, both male and females responded to the treatment and no sex differences were observed in the majority of the tested parameters.

SE-induced long-term effects on the brain structure and functions in experimental models of epilepsy are well known [21, 36, 44]. Soman-induced long-term effects, especially the changes in the hippocampus and amygdala, have been demonstrated in rat models [36, 45–48]. Some interventional studies such as targeting the GluK1/AMPA receptors with LY293558, or muscarinic receptors with caramiphen, have demonstrated the mitigating effects in soman-induced brain pathology and dysfunction in a 4-week study [49, 50]. A combination of antiseizure medications such as phenobarbital, ketamine, and midazolam demonstrated a reduction in soman‐induced epileptogenesis and brain pathology in a rat model in a 2-week study [51, 52]. In another study, treating guinea pigs with galantamine, a glutamate antagonist, significantly reduced soman-induced lethality and mitigated hippocampal-dependent memory deficits [53]. In all these models, atropine, an anticholinergic and an oxime (HI-6 and/or 2-PAM) were administered immediately after soman exposure to control mortality. Atropine has a narrow window of action, therefore, should be administered no later than 20 min of exposure. The oximes do not effectively penetrate the brain to protect it from SE-induced effects [9, 54]. However, none of these countermeasures protect the brain from acute exposure to nerve agents. In addition to these and ion channel-targeted drugs, our recent studies demonstrate the role of nitrooxidative stress and iNOS as a potential target for disease modification in DFP-induced models of epilepsy [14, 55]. Antioxidant drug therapy as a neuroprotective countermeasure for nerve agent toxicity has been proposed in another study [56].

Soman-induced SE in experimental models, depending on the severity, leads to the development of epilepsy and behavioral dysfunction [46, 57–59]. In soman-exposed animals, the total time traveled in the open field was significantly higher than their respective controls suggesting the hyper locomotor activity and anxiety-like behavior. However, when they were subjected to accelerated motor skill test (rotarod), vehicle-treated soman-exposed animals demonstrated a significant deficiency which was rescued by 1400W, implying the role of iNOS/nitrooxidative stress-mediated pathway in skilled motor activity. Interestingly, no sex interaction was observed in any of the behavioral studies.

The role of amygdala, the bed nucleus of stria terminalis (BNST), the ventral hippocampus (“anxiety cells”) and their projections to hypothalamus and brain stem mediate fear memory and anxiety-like behaviors [60–63]. The amygdala is highly susceptible to OPNA exposure and causes loss of inhibitory neurons and seizures, which, in the long-term, induces neurodegeneration and the development of anxiety-like behavior in rodent models [8, 49, 64–67]. The occurrence of SRS in soman model in this study could be due to the loss of inhibitory neurons in the amygdala and its consequence of hyperexcitability [47, 68–70]. In a related soman exposure study in our laboratory, functional brain MRI revealed a significant loss of network connectivity in the hippocampus and cortex suggesting that the role of these networks too in mediating seizures onset and/or propagation. Stereological cell count confirmed a significant reduction in neuronal population in amygdala and the piriform cortex in the soman-exposed animals (Fig. 8D). 1400W treatment did not completely prevent neuronal loss in these regions. However, when parvalbumin (PVB) positive neurons were counted from the amygdala, we found a significant protection by 1400W (Fig. 8E). Considering the ratio of inhibitory and excitatory neuronal populations, rescue of a small number of inhibitory neurons could dampen the overall excitability in the network. We observed a significant reduction of compromised anxiety/fear-like behavior in the elevated zero/circular maze test compared to the vehicle treated group in the soman-exposed animals (Fig. 3B, C) implying that the survival of certain subsets of inhibitory neurons, such as PVB, in the amygdala by 1400W may have mitigated the soman-induced effects. Interestingly, there were no differences in the total distance traveled and the time spent at the center in the open field test in 1400W and vehicle-treated soman-exposed animals, which may suggest that 1400W be effective in mitigating certain types of fear-associated pathways. However, the impact of soman on BNST and its projection fibers to the other brain regions are not investigated in this study.

Freezing behavior is a physiological response to a stimulus and a measure of fear in experimental models [59, 71, 72]. Injury to amygdala in rodent model prevented fear-potentiated freezing/startle response to foot-shock when paired with exposure to bright light or a tone [73–75]. The contextual fear conditioning response reveals functional network connectivity between the hippocampus (contextual recollection), amygdala (fear/anxiety-like behavior), BNST, brain stem and hypothalamus [61, 63]. If the tone is introduced as a context followed by foot shock, as in this study, the auditory pathway will be activated upon the introduction of the tone in a normal individual. Injury to any of these structures will yield a different response which is measured as the increased duration of freezing following a tone as in this study. Initial SE severity determines the extent of injury to various brain regions. For example, hippocampus is less likely be affected compared to the basolateral amygdala and the piriform cortex if initial SE persists for ~ 20 min after acute exposure in OPNA model. If SE severity is > 20 min, widespread brain pathology occurs [14, 21]. In this study, SE severity was > 20 min. Therefore, it was anticipated that the animals exposed to soman and 1400W would behave differently. All animals responded similarly to the tone and the foot shock during the conditioning phase (Fig. 3G) suggesting the response pathway to noxious stimulus (fear learning) was unaffected in soman-exposed animals. However, the obvious differences between groups emerged when they were probed 24 h post-conditioning without foot shock implying the disruption in the connectivity between amygdala and the other brain regions. In an another soman exposure study, male rats revealed impaired auditory and contextual fear conditioning due to neurodegeneration in the piriform cortex, amygdala, thalamus, and the hippocampus [59]. Our present study confirmed pathology in some of these brain regions. 1400W reduced neurodegeneration in the centromedial (in males) and mediodorsal (in females) thalamic nuclei, and amygdala (increased PVB neurons) (Figs. 7D, E and 8E). However, we did not find sex differences in behavioral outcomes and in the effects of 1400W on PVB neurons.

Loss of inhibitory neurons or functional connectivity due to soman exposure is known to cause hyperexcitability in the network and onset of seizures [46, 47]. The epileptiform spiking and SRS frequency in soman-exposed vehicle-treated males was significantly reduced by 1400W in soman-exposed animals suggesting the disease-modifying potential of the iNOS inhibitor. We have previously demonstrated similar disease-modifying effects of 1400W in both DFP and KA rat models of chronic epilepsy [14, 20]. In a rat neuropathic pain model, 1400W significantly reduced pain sensitivity [76]. In an in vitro brain slice model too, pre-treating with 1400W significantly reduced KA-induced hyperexcitability [77]. In contrast, in females exposed to soman, no significant effects of 1400W on EEG parameters were observed in this study. This could be due to the secondary effects of the subcutaneously implanted transmitter devices that caused skin ulcers (bed sores) in chronic studies in females due to the thin skin. This resulted in repeated repositioning of the transmitter device under brief general anesthesia (isoflurane). Repeated isoflurane exposures in the rat model impaired long-term potentiation (LTP) at both 1 day and 1 week after the last exposure, which lasted for about a month [78]. In both KA and paraoxon rat model, SE induction in the presence of isoflurane did not affect the severity of SE but impacted epileptogenesis [79]. In this study, since female rats were exposed more often to isoflurane than males, there was an overall reduction in SRS frequency in both vehicle and 1400W treated groups (Fig. 10I), therefore, isoflurane exposure and frequent repositioning of transmitter device may have confounded the SRS frequency and the real effect of 1400W in females.

Nitrooxidative stress and proinflammatory cytokines and chemokines production are well-known mediators of epileptogenesis that follows chemoconvulsant-induced SE [14, 37, 56, 80, 81]. In OPNA toxicity models, we also demonstrated the roles of two RNS/ROS generating enzymes, the iNOS and the NADPH oxidase 2 (NOX2). These enzymes and the nitrosylated (3NT) or glutathiolated (anti-GSH) proteins were significantly upregulated in the rat brain following exposure to an OPNA. Additionally, we observed a significant increase in reactive gliosis (C3-positive astrocytes and CD68-positive microglia) and neurodegeneration in several brain regions in DFP or soman-exposed animals [1, 14, 21]. Reactive glial cells are known to produce proinflammatory cytokines and iNOS [14, 82, 83]. Treating with an iNOS inhibitor (1400W) or a NOX2 inhibitor (Diapocynin) mitigated the DFP-induced nitrooxidative stress, neuroinflammation, neurodegeneration, and neuronal hyperexcitability [14, 55]. These findings suggest that the antioxidant mechanism is a potential therapeutic target for disease modification in OPNA models.

It is interesting to note that there are regional variation and sex differences in reactive microglia that contained CD68 and reactive astrocytes with C3, which may have had impacted the behavioral outcome. Significant reduction of reactive microglia by 1400W in soman-exposed group was observed in amygdala and thalamus of females but not males. However, no sex interaction was observed in the suppression of CD68-positive reactive microglia by 1400W in these regions. This may suggest that it is unlikely that a mere reduction of reactive microglia alone determines the functional outcome. The other parameters such as GFAP + C3, inhibitory neuronal recovery, and functional network connectivity could mediate the process. Reactive microglia more frequently contain CD68 [31, 32]. CD68 is a lysosomal transmembrane glycoprotein expressed in high levels in phagocytic microglia. In contrast, reactive astrocytes express C3 [33, 34]. In the brain, C3 is a mediator of innate immunity and communicate with microglia for synaptic pruning during development [84–86]. In injured brain, reactive astrocytes release C3, which we found in the extra cellular spaces, and could reactive microglia, which express receptors for C3 [34]. Although we did not investigate the extent of C3a receptors expression in microglia, increased expression of CD68 in vulnerable brain regions such as AMY, PC, and thalamic nuclei suggest the ongoing phagocytosis. Furthermore, in these regions there was a significant increase in neurodegeneration, especially the parvalbumin-positive inhibitory neurons in amygdala, in soman-exposed animals that was reduced by 1400W. Loss of such inhibitory neurons, reactive gliosis and a concurrent increase in cytokines and nitrooxidative stress may have caused neuronal hyperexcitability and spontaneously recurring seizures in soman-exposed animals. Treating with 1400W reduced soman-induced effects.

This study encompasses a rigorous investigation of sex differences across multiple analyses by incorporating a factorial design of both sex and treatment effects [30]. We examined the interaction effects of sex on treatment outcomes, rather than disaggregating the data by sex and then examining for treatment effects, as has traditionally been the norm in previous studies. Such robust studies yield meaningful interpretations of true sex differences in translational outcomes for preclinical studies.

In summary, the MCM treatment (atropine, oxime, midazolam) alone had no long-term protective effect in soman-induced SE in both males and females. 1400W treatment for two weeks mitigated soman-induced motor and memory deficits, and reduced anxiety/fear-like behavior. No sex differences were observed in behavioral tests. 1400W also significantly reduced epileptiform spike rate (a measure of neuronal hyperexcitability) and SRS frequency in males but not in females. Nitrooxidative markers such as nitrite, ROS, and oxidized glutathione, and proinflammatory cytokines and chemokine in the serum and CSF were significantly reduced in 1400W treated group. Additionally, the soman-induced reactive gliosis was reduced by 1400W treatment in some brain regions in both sexes. Parvalbumin positive neurons were protected by 1400W in amygdala in soman-exposed animals. Overall, these findings suggest the disease-modifying effects of a highly selective iNOS inhibitor, 1400W, in an experimental model of nerve agent neurotoxicity.

## Supplementary Information

**Additional file 1: Table S1**. The list of antibodies and reagents used in the experiments. **Table S2.** Details of statistical analyses applied for each experiment/figure.

## Data Availability

All the raw data and processed data are stored in University’s long-term data storage facility and can be accessed by requesting tswamy@iastate.edu. This project did not generate any materials to share. All the materials are available for purchase through commercial companies which is stated in the manuscript.
