# Supplementary material for: Disease-modifying effects of a glial-targeted inducible nitric oxide synthase inhibitor (1400W) in mixed-sex cohorts of a rat soman (GD) model of epilepsy
Source: J Neuroinflammation. 2023 Jul 12;20:163. doi: 10.1186/s12974-023-02847-1 (PMC10337207; doi:10.1186/s12974-023-02847-1)
Supplement: Supplementary file 1 — Additional file 1: Table S1. The list of antibodies and reagents used in the experiments. Table S2. Details of statistical analyses applied for each experiment/figure. [file 12974_2023_2847_MOESM1_ESM.docx]

**Supplementary Table 1. Antibodies and reagents used in experiments**

| **Primary Antibody** | **Source** | **Catalogue number** | **Dilution factor** |
| --- | --- | --- | --- |
| Anti-NeuN (rabbit) | EMD Millipore | ABN78 | 1:200 |
| Anti-IBA1 (goat) | Abcam | Ab5076 | 1:300 |
| Anti-GFAP (mouse) | Sigma Aldrich | G3893 | 1:300 |
| Anti-C3 (rat) | Novus | NB200-540 | 1:50 |
| Anti-CD68 (Rabbit) | Abcam | Ab125212 | 1:400 |
| Anti-Parvalbumin (Rabbit) | Abcam | Ab11427 | 1:1000 |
| **Secondary Antibody** | **Source** | **Catalogue number** | **Dilution factor** |
| Rhodamine Red™-X anti-rabbit | Jackson ImmunoResearch | 111-295-144 | 1:200 |
| Rhodamine Red™-X anti-goat | Jackson ImmunoResearch | 705-295-147 | 1:200 |
| Alexa Fluor® 488 anti-rabbit | Jackson ImmunoResearch | 711-545-152 | 1:200 |
| Biotinylated Donkey anti-rat | Jackson ImmunoResearch | 712-065-153 | 1:100 |
| Alexa Fluor® 488 anti-mouse | Jackson ImmunoResearch | 115-545-003 | 1:300 |
| Cy^TM^3-conjugated Streptavidin | Jackson ImmunoResearch | 016-160-084 | 1:100 |

**Supplementary Table 2. Details of statistical analysis applied for each experiment/figure.**

| **Fig. Panel** | **Graph description** | **Control** | **Veh+ 1400W** | **Soman+ Veh** | **Soman+ 1400W** | **males** | **females** | **Sex interaction (p-value)** | **Statistical test** |
| --- | --- | --- | --- | --- | --- | --- | --- | --- | --- |
| Figure 2  (A) | Rotarod | 24 | 25 | 25 | 26 | 11-13 | 12-13 | 0.7946 | Tukey's multiple comparisons test |
| Figure 2  (E) | NOR (3hrs) | 19 | 19 | 16 | 21 | 8-13 | 8-11 | 0.149 | Tukey's multiple comparisons test |
| Figure 2  (F) | NOR (24hrs) | 24 | 26 | 23 | 25 | 11-14 | 9-13 | 0.534 | Tukey's multiple comparisons test |
| Figure 3  (C) | Zero Maze | 24 | 25 | 27 | 28 | 11-15 | 12-13 | 0.1542 | Tukey's multiple comparisons test |
| Figure 3  (D) | Open Field (Center) | 24 | 26 | 27 | 27 | 11-15 | 12-13 | 0.5868 | Tukey's multiple comparisons test |
| Figure 3  (E) | Open Field (Periphery) | 24 | 26 | 27 | 27 | 11-15 | 12-13 | 0.5866 | Tukey's multiple comparisons test |
| Figure 3 (F) | Open Field (Distance Travelled) | 24 | 26 | 27 | 27 | 11-15 | 12-13 | 0.2256 | Tukey's multiple comparisons test |
| Figure 3  (G, H) | Contextual Fear  Conditioning (Conditioning & Probe) | 24 | 26 | 27 | 27 | 11-15 | 12-13 | NA | Tukey's multiple comparisons test |
| Figure 3  (I) | Freezing Time (%) Increase | 24 | 25 | 27 | 27 | 11-15 | 12-13 | 0.4092 | Tukey's multiple comparisons test |
| Figure 4  (A) | Nitrite | 7 | 8 | 8 | 8 | 3-4 | 4 | 0.9431 | Tukey's multiple comparisons test |
| Figure 4  (B) | ROS | 8 | 6 | 7 | 7 | 2-4 | 4 | NA | Tukey's multiple comparisons test |
| Figure 4  (C) | GSH/GSSG | 8 | 0 | 8 | 8 | 4 | 4 | 0.9506 | Tukey's multiple comparisons test |
| Figure 4  (D) | IL1-β (Serum) | 7 | 0 | 7 | 7 | 4 | 3 | 0.9532 | Tukey's multiple comparisons test |
| Figure 4  (D) | TNF-α (Serum) | 7 | 0 | 7 | 7 | 4 | 3 | 0.7731 | Tukey's multiple comparisons test |
| Figure 4  (D) | MCP-1 (Serum) | 7 | 0 | 7 | 7 | 4 | 3 | 0.8843 | Tukey's multiple comparisons test |
| Figure 4  (D) | IL-6 (Serum) | 8 | 0 | 8 | 7 | 3-4 | 4 | 0.9509 | Tukey's multiple comparisons test |
| Figure 4  (E) | IL1-β (CSF) | 5 | 0 | 7 | 7 | NA | NA | NA | Tukey's multiple comparisons test |
| Figure 4  (E) | TNF-α (CSF) | 5 | 0 | 7 | 7 | NA | NA | NA | Tukey's multiple comparisons test |
| Figure 4  (E) | MCP-1 (CSF) | 5 | 0 | 7 | 7 | NA | NA | NA | Dunn's multiple comparisons test |

| **Fig. Panel** | **Graph description** | **Region** | **Control** | **Veh+1400W** | **Soman+Veh** | **Soman+1400W** | **males** | **females** | **Sex interaction (p-value)** | **Statistical test** |
| --- | --- | --- | --- | --- | --- | --- | --- | --- | --- | --- |
| Figure 5 (D) | Microglia | DG | 9 | 10 | 9 | 8 | 3-5 | 5 | 0.3782 | Tukey's multiple comparisons test |
|  |  | CA3 | 9 | 10 | 9 | 8 | 3-5 | 5 | 0.4208 | Tukey's multiple comparisons test |
|  |  | CA1 | 9 | 10 | 9 | 8 | 3-5 | 5 | 0.383 | Tukey's multiple comparisons test |
|  |  | SUB | 9 | 10 | 9 | 8 | 3-5 | 5 | 0.2105 | Tukey's multiple comparisons test |
|  |  | AMY | 9 | 10 | 9 | 8 | 3-5 | 5 | 0.0487 | Tukey's multiple comparisons test |
|  |  | PC | 9 | 10 | 9 | 8 | 3-5 | 5 | 0.049 | Tukey's multiple comparisons test |
|  |  | LDT | 9 | 10 | 9 | 8 | 3-5 | 5 | 0.0707 | Tukey's multiple comparisons test |
|  |  | MDT | 9 | 10 | 9 | 8 | 3-5 | 5 | 0.112 | Tukey's multiple comparisons test |
|  |  | CMT | 9 | 10 | 9 | 8 | 3-5 | 5 | 0.0797 | Tukey's multiple comparisons test |
| Figure 5 (H) | Reactive Microglia | DG | 9 | 10 | 9 | 8 | 3-5 | 5 | 0.4221 | Tukey's multiple comparisons test |
|  |  | CA3 | 9 | 10 | 9 | 8 | 3-5 | 5 | 0.1637 | Tukey's multiple comparisons test |
|  |  | CA1 | 9 | 10 | 9 | 8 | 3-5 | 5 | 0.2028 | Tukey's multiple comparisons test |
|  |  | SUB | 9 | 10 | 9 | 8 | 3-5 | 5 | 0.1838 | Tukey's multiple comparisons test |
|  |  | AMY | 9 | 10 | 9 | 8 | 3-5 | 5 | 0.004 | Tukey's multiple comparisons test |
|  |  | PC | 9 | 10 | 9 | 8 | 3-5 | 5 | 0.4825 | Tukey's multiple comparisons test |
|  |  | LDT | 9 | 10 | 9 | 8 | 3-5 | 5 | 0.0389 | Tukey's multiple comparisons test |
|  |  | MDT | 9 | 10 | 9 | 8 | 3-5 | 5 | <0.0001 | Tukey's multiple comparisons test |
|  |  | CMT | 9 | 10 | 9 | 8 | 3-5 | 5 | 0.0057 | Tukey's multiple comparisons test |
| Figure 5 (M) | IBA1-CD68 | AMY | 9 | 10 | 9 | 8 | 3-5 | 5 | 0.1484 | Tukey's multiple comparisons test |
|  |  | PC | 9 | 10 | 9 | 8 | 3-5 | 5 | 0.1545 | Tukey's multiple comparisons test |
|  |  | LDT | 9 | 10 | 9 | 8 | 3-5 | 5 | 0.1327 | Tukey's multiple comparisons test |
|  |  | MDT | 9 | 10 | 9 | 8 | 3-5 | 5 | 0.0946 | Tukey's multiple comparisons test |
|  |  | CMT | 9 | 10 | 9 | 8 | 3-5 | 5 | 0.5771 | Tukey's multiple comparisons test |
| Figure 6 (D) | Astroglia | DG | 9 | 10 | 9 | 8 | 3-5 | 5 | 0.28 | Tukey's multiple comparisons test |
|  |  | CA3 | 9 | 10 | 9 | 8 | 3-5 | 5 | 0.8589 | Tukey's multiple comparisons test |
|  |  | CA1 | 9 | 10 | 9 | 8 | 3-5 | 5 | 0.4342 | Tukey's multiple comparisons test |
|  |  | SUB | 9 | 10 | 9 | 8 | 3-5 | 5 | 0.1353 | Tukey's multiple comparisons test |
|  |  | AMY | 9 | 10 | 9 | 8 | 3-5 | 5 | 0.9925 | Tukey's multiple comparisons test |
|  |  | PC | 9 | 10 | 9 | 8 | 3-5 | 5 | 0.4832 | Tukey's multiple comparisons test |
|  |  | LDT | 9 | 10 | 9 | 8 | 3-5 | 5 | 0.3594 | Tukey's multiple comparisons test |
|  |  | MDT | 9 | 10 | 9 | 8 | 3-5 | 5 | 0.7526 | Tukey's multiple comparisons test |
|  |  | CMT | 9 | 10 | 9 | 8 | 3-5 | 5 | 0.2993 | Tukey's multiple comparisons test |
| Figure 6 (F) | Reactive Astroglia | DG | 9 | 10 | 9 | 8 | 3-5 | 5 | 0.1062 | Tukey's multiple comparisons test |
|  |  | CA3 | 9 | 10 | 9 | 8 | 3-5 | 5 | 0.1352 | Tukey's multiple comparisons test |
|  |  | CA1 | 9 | 10 | 9 | 8 | 3-5 | 5 | 0.2169 | Tukey's multiple comparisons test |
|  |  | SUB | 9 | 10 | 9 | 8 | 3-5 | 5 | 0.1035 | Tukey's multiple comparisons test |
|  |  | AMY | 9 | 10 | 9 | 8 | 3-5 | 5 | 0.5498 | Tukey's multiple comparisons test |
|  |  | PC | 9 | 10 | 9 | 8 | 3-5 | 5 | 0.3891 | Tukey's multiple comparisons test |
|  |  | LDT | 9 | 10 | 9 | 8 | 3-5 | 5 | 0.1269 | Tukey's multiple comparisons test |
|  |  | MDT | 9 | 10 | 9 | 8 | 3-5 | 5 | 0.0733 | Tukey's multiple comparisons test |
|  |  | CMT | 9 | 10 | 9 | 8 | 3-5 | 5 | 0.0404 | Tukey's multiple comparisons test |
| Figure 6 (J) | GFAP-C3 | DG | 9 | 10 | 9 | 8 | 3-5 | 5 | 0.0955 | Tukey's multiple comparisons test |
|  |  | CA3 | 9 | 10 | 9 | 8 | 3-5 | 5 | 0.6213 | Tukey's multiple comparisons test |
|  |  | CA1 | 9 | 10 | 9 | 8 | 3-5 | 5 | 0.2895 | Tukey's multiple comparisons test |
|  |  | SUB | 9 | 10 | 9 | 8 | 3-5 | 5 | 0.861 | Tukey's multiple comparisons test |
|  |  | AMY | 9 | 10 | 9 | 8 | 3-5 | 5 | 0.1279 | Tukey's multiple comparisons test |
|  |  | PC | 9 | 10 | 9 | 8 | 3-5 | 5 | 0.1011 | Tukey's multiple comparisons test |
|  |  | LDT | 9 | 10 | 9 | 8 | 3-5 | 5 | 0.8596 | Tukey's multiple comparisons test |
|  |  | MDT | 9 | 10 | 9 | 8 | 3-5 | 5 | 0.6128 | Tukey's multiple comparisons test |
|  |  | CMT | 9 | 10 | 9 | 8 | 3-5 | 5 | 0.5773 | Tukey's multiple comparisons test |
| Figure 7 (C) | FJB+ve NeuN Cells | DG | 9 | 10 | 9 | 9 | 3-5 | 5-6 | 0.6956 | Tukey's multiple comparisons test |
|  |  | CA3 | 9 | 10 | 9 | 9 | 3-5 | 5-6 | 0.865 | Tukey's multiple comparisons test |
|  |  | CA1 | 9 | 10 | 9 | 9 | 3-5 | 5-6 | 0.4079 | Tukey's multiple comparisons test |
|  |  | SUB | 9 | 10 | 9 | 9 | 3-5 | 5-6 | 0.0473 | Tukey's multiple comparisons test |
|  |  | AMY | 9 | 10 | 9 | 9 | 3-5 | 5-6 | 0.0291 | Tukey's multiple comparisons test |
|  |  | PC | 9 | 10 | 9 | 9 | 3-5 | 5-6 | 0.2074 | Tukey's multiple comparisons test |
|  |  | LDT | 9 | 10 | 9 | 9 | 3-5 | 5-6 | 0.2381 | Tukey's multiple comparisons test |
|  |  | MDT | 9 | 10 | 9 | 9 | 3-5 | 5-6 | 0.0087 | Tukey's multiple comparisons test |
|  |  | CMT | 9 | 10 | 9 | 9 | 3-5 | 5-6 | 0.0328 | Tukey's multiple comparisons test |
| Figure 8 (D) | Stereology | CA1 | 8 | NA | 8 | 8 | NA | NA | NA | Tukey's multiple comparisons test |
|  |  | Hilus | 8 | NA | 8 | 8 | NA | NA | NA | Dunn's multiple comparisons test |
|  |  | PC | 7 | NA | 10 | 8 | NA | NA | NA | Dunn's multiple comparisons test |
| Figure 8 (E) | Parvalbumin | AMY | 9 | 10 | 8 | 10 | 3-5 | 4-7 | 0.0126 | Tukey's multiple comparisons test |
